# Forming attitudes via neural activity supporting affective episodic simulations

**DOI:** 10.1038/s41467-019-09961-w

**Published:** 2019-05-17

**Authors:** Roland G. Benoit, Philipp C. Paulus, Daniel L. Schacter

**Affiliations:** 1 Max Planck Institute for Human Cognitive and Brain Sciences, Leipzig, 04103 Germany; 2 International Max Planck Research School NeuroCom, Leipzig, 04103 Germany; 3 Department of Psychology, Harvard University, Cambridge, MA 02138 USA

**Keywords:** Cognitive neuroscience, Psychology

## Abstract

Humans have the adaptive capacity for imagining hypothetical episodes. Such episodic simulation is based on a neural network that includes the ventromedial prefrontal cortex (vmPFC). This network draws on existing knowledge (e.g., of familiar people and places) to construct imaginary events (e.g., meeting with the person at that place). Here, we test the hypothesis that a simulation changes attitudes towards its constituent elements. In two experiments, we demonstrate how imagining meeting liked versus disliked people (unconditioned stimuli, UCS) at initially neutral places (conditioned stimuli, CS) changes the value of these places. We further provide evidence that the vmPFC codes for representations of those elements (i.e., of individual people and places). Critically, attitude changes induced by the liked UCS are based on a transfer of positive affective value between the representations (i.e., from the UCS to the CS). Thereby, we reveal how mere imaginings shape attitudes towards elements (i.e., places) from our real-life environment.

## Abstract (web summary)

People vividly simulate prospective events and experience the anticipated affect—processes supported by the ventromedial prefrontal cortex (vmPFC). Here, the authors show that these mere simulations change real-life attitudes, via a value transfer between environmental representations in the vmPFC.

## Introduction

A remarkable feat of the human mind is its ability to vividly imagine a plethora of prospective events^1,2^. The core brain network supporting such episodic simulations comprises parts of the medial surface, including the ventromedial prefrontal cortex (vmPFC), lateral parts of the inferior posterior and temporal cortices, and the medial temporal lobes^1,3,4^. This network has been suggested to mediate episodic simulation by supporting the integration of elements from disparate episodic and semantic memories (e.g., of a liked person and a neutral but hitherto unrelated place) into novel events (e.g., meeting that person at that place for the first time)^1,2,5,6^.

Simulating prospective events influences how we anticipate the future, for example by conveying the anticipated affect of an imagined event^7,8^. Here, we examine the hypothesis that it also changes how we value our immediate present by shaping real-life attitudes.

Episodic simulation creates an imaginary parallel to a situation of actually pairing a valenced unconditioned stimulus (UCS) with an initially neutral conditioned stimulus (CS). Such evaluative conditioning forms attitudes by changing the liking of the CS to align with the valence of the UCS^9–11^. We hypothesize that imaginings of possible events (e.g., meeting a beloved person at a specific place) can effectively transfer affective value from one of the integrated elements (e.g., the person) to the other (e.g., the place). By this process, episodic simulations modify attitudes toward the very elements that the simulations had been based on, thus influencing how we evaluate our real-life environment.

Our hypothesis assigns a key role to the vmPFC in mediating such presumed attitude change. This region has been shown to integrate similar memories into schematic representations of the elements that are shared across those memories^12–15^. The concurrent reactivation of disparate representations, in turn, can support simulations of even novel experiences^16,17^.

Critically, the vmPFC does not only represent “cool” models of the world^18^. Activation in this region also generally varies with subjective value^19,20^, and it specifically scales with the affective quality of simulated experiences^16,17,21,22^. The vmPFC has thus been associated with both schematic knowledge and the representation of affective value. During episodic simulation, these two functions are supported by overlapping parts of the vmPFC^16,17,23^^,*see also*^^24^. This overlap is consistent with the hypothesis that this region codes for schematic representations that also entail associated affect, i.e., for “hot” models of the world^18,25^.

The vmPFC may thus code for affective representations of elements from our environment that can be flexibly integrated to support affective episodic simulations. Here, we hypothesize that such simulation-based integration induces experience-dependent plasticity in the neuronal coding of the individual elements^16^. This plasticity could then enable the transfer of affective value from one element (i.e., the UCS) of the episode to the other (i.e., the CS).

**Fig. 1:**
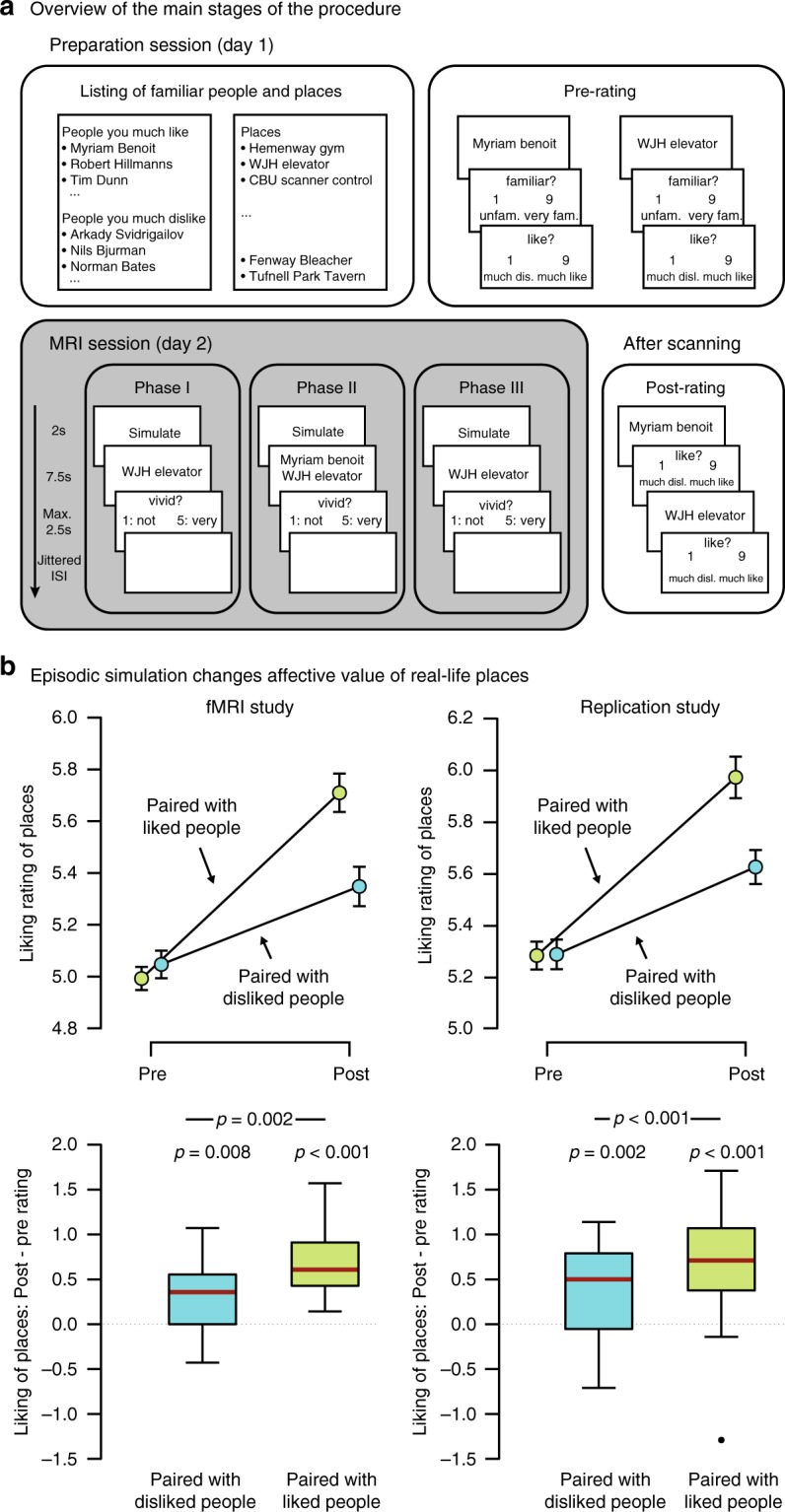
Main stages of the procedure and behavioral results. **a** In an initial session, participants provided names of both liked and disliked people as well as of specific places from their everyday environment. They then rated how much they liked the people and places (indexing value) and how familiar they are with each of those. Based on the ratings, we selected neutral places and combined each of those with either a liked or a disliked person. In a second session, participants were scanned with fMRI during three phases: In phase I, they imagined interacting with each person and place in isolation. In phase II, they were shown the critical pairings and imagined interacting with the respective person in a way that would be specific to that place. Phase III was identical to phase I, except for a different presentation order. Finally, outside the scanner, participants re-rated their liking of each person and place. Moreover, they indicated, for each person–place pairing, the plausibility of such a meeting and the anticipated pleasantness. **b** Consistent across a fMRI study and a pre-registered replication study, we observed that places were deemed more positive following the integrative simulations. Critically, this pattern was stronger for places that had been the imaginary locations of meetings with liked than with disliked people. Episodic simulation thus induced a change in attitude of the UCS that was contingent on the valence of the CS. Error bars in the pre- versus post panels indicate the respective standard error of the mean. Boxplots indicate the median, central quartiles, and + /− 2.7 SD. The dot denotes an outlier beyond that range

To test this hypothesis, we combined a novel experimental procedure with functional MRI (fMRI) and representational-similarity analysis^26^ (Fig. 1a). Before the fMRI session, participants provided names of places and people that they personally knew. For the latter, we specified that participants should name both people that they much liked, as well as those that they much disliked. They then rated the familiarity and liking (as an index of value) of each place and person (pre-rating). Based on these ratings, we selected places that the participants felt neutral towards (i.e., the CS) and paired these with either the most liked or much disliked people (i.e., the positive and negative UCS).

These elements and their pairings then featured during the three phases of the fMRI session (Fig. 1a). During phase I, we presented each person and place, one at a time (i.e., the items were not presented as pairs during this phase), and participants vividly imagined interacting with the given person or acting in a way that would be typical for the location. During phase II, they encountered each person/place pairings repeatedly, and their task was to imagine interacting with the person in a location-specific manner. These simulations thus required the integration of the two-paired elements. Phase III repeated phase I with a different presentation order. Finally, outside the scanner, participants rated the liking of each person and place again (post rating) before they indicated the plausibility of meeting a given person at its paired location as well as the anticipated pleasantness of such an event.

This procedure allowed us to test the predicted impact of affective simulations on real-life attitudes in the fMRI study and in a behavioral replication study. The results from the two studies indicate that simulations indeed cause a positive change in attitudes, particularly toward those places that had been the location for imaginary meetings with liked people. At the same time, the fMRI results support key predictions of the neural basis of this effect. We first provide evidence for the premise of our hypothesis that the vmPFC codes for affective representations of individual elements from everyday life: episodic simulations yield replicable activity patterns that are specific for individual people and places. The activation in this region moreover reflects the affective value of the respective element. We then provide evidence for our proposal that the attitude change (at least for places imagined with liked people) is mediated by a transfer of affective value: during integrative simulations, vmPFC activation reflects the value of the person (i.e., the UCS) and predicts the ensuing shift in attitude toward the paired place (i.e., the CS). The current data thus demonstrate how imaginings, much like real happenings, can have a powerful influence on our attitudes and, thereby, shape our models of the world.

## Results

### Overview

In the following, we first establish whether simulated episodes, similar to actual encounters^9,10^, can shape real-life attitudes by reporting the behavioral results of an fMRI study (*n* = 18) and a pre-registered replication (*n* = 30). We then examine the complementary hypothesis regarding the involvement of the vmPFC.

### Episodic simulation changes real-life attitudes

Based on the pre-ratings, we had paired each neutral place with either a liked or disliked person (difference in liking: *t*_17 _= 47.2, *p* < 0.001, *d* = 11.13). The liked people, however, were also more familiar than the disliked people (difference in familiarity rating: mean = 2.23, standard error = 0.35; *t*_17 _= 6.47, *p* < 0.001, *d* = 1.53), while the places in the two conditions were well matched on this dimension (*t*_17 _= −0.46, *p* = 0.65, *d* = −0.11) (Supplementary Table 1).

Critically, in phase II, participants then repeatedly imagined interactions with each person at their respective paired place. Participants experienced episodes featuring liked people as more plausible (*t*_17 _= 3.19, *p* = 0.005, *d* = 0.75) and, importantly, also as more pleasant (*t*_17 _= 15.88, *p* < 0.001, *d* = 3.74). Mentally integrating elements into a common episode thus elicited an affective experience that was aligned with the valence of the UCS.

We predicted that the affective experience, in turn, would change attitudes toward the episodes’ (initially neutral) locations. In particular, there should be a greater increase in liking for places that had been the stage for imaginary meetings with liked than with disliked people. We quantified the change in liking by computing the difference scores of the post- and pre-rating. Though these scores indicated that both kinds of places were deemed more positive following any simulation (paired with liked people: *t*_17 _= 7.9, *p* < 0.001, *d* = 1.86; paired with disliked people: *t*_17 _= 3.043, *p* = 0.008, *d* = 0.71), this shift in attitude was indeed greater for places that had been imagined with liked people (*t*_17 _= 3.68, *p* = 0.002, *d* = 0.87) (Fig. 1b) (for concomitant changes in the attitudes toward the people, see Supplementary Fig. 1).

Given that the liked and disliked people also differed in familiarity, we examined the change in liking while controlling for this possible confound. In particular, for each difference score, we first regressed out the effect of familiarity. We then examined the ensuing residual scores, which were indeed still larger for the places imagined with liked than with disliked people (due to a deviation from normality, as indicated by Shapiro–Wilk, *W* = 0.831, *p* = 0.004, tested with a Wilcoxon test: *W*_17_ = 143, *p* = 0.005, matched rank-biserial correlation, *r* = 0.67). This result thus indicates that the attitude change toward the places was based on the valence of the paired people rather than on their familiarity.

### The simulation-induced attitude change is replicable

Due to the novelty of this behavioral effect, we sought to determine its replicability by running a pre-registered study (https://aspredicted.org/9ti3h.pdf). The procedure was identical to the fMRI study, except for the omission of phases I and III. We also employed a modified algorithm that successfully matched the selected liked and disliked people on familiarity (Supplementary Methods and Supplementary Table 2). Critically, this study yielded the identical pattern of a more positive change in liking for places that had been imagined with liked rather than with disliked people (*t*_29 _= 3.77, *p* < 0.001, *d* = 0.69) (Supplementary Note; Fig. 1b).

The mere act of imagining interactions can thus change real-life attitudes. In the following, we examine the hypothesis that such changes are mediated by a transfer of affective value between neural representations in the vmPFC. We tested three key predictions: first, a premise of the hypothesis is that neurons in the vmPFC code for representations of elements from our environment. Second, it posits that these representations entail information about the elements’ affective value. Finally, the hypothesis proposes that the vmPFC mediates attitude changes by transferring affective value from the UCS (i.e., the person) to the CS (i.e., the place).

### vmPFC codes for individual people and places

First, if the vmPFC codes for individual representations, then activation in the vmPFC should carry information about the identity of specific people and places. We tested this prediction by examining the replicability of simulation-induced activity patterns from phase I to phase III using representational-similarity analysis (RSA)^26^.

Neuronal representations are assumed to be reflected in distributed activity patterns that can be assessed with fMRI^26,27^. Thus, to the degree that a specific representation is engaged whenever one imagines a particular person (or place), a similar activity pattern should get re-instated whenever one simulates an episode featuring the same person (or the same place). Accordingly, activity patterns should be more similar for the comparison of a given element with its repetition (within-item similarity) than with a different element at the time of the repetition (between-item similarity)^27^. Moreover, if the activity pattern truly reflects the neural representation of a given element (e.g., a particular liked person)—rather than just category membership (e.g., all people) or valence (e.g., all liked elements)—we expect the within-item similarity to be greater even when restricting the between-item similarity to elements of the same category (e.g., only other liked people).

**Fig. 2:**
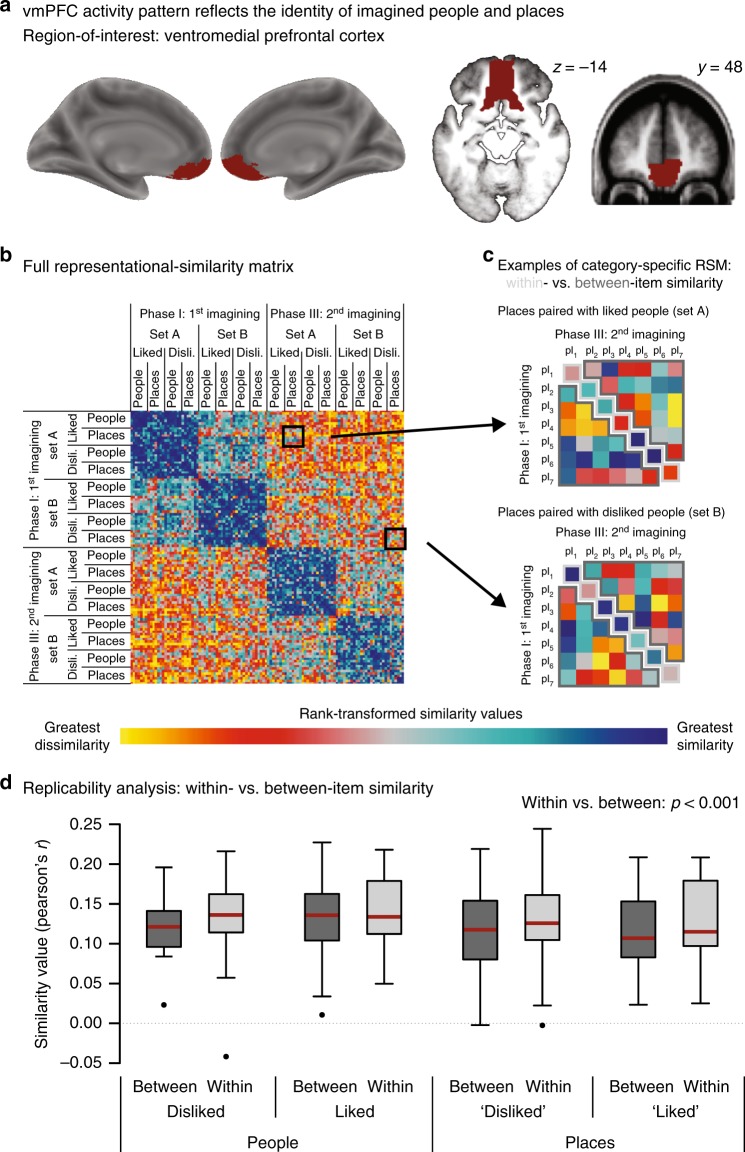
Representational-similarity analysis (RSA) yields environmental representations in the vmPFC. **a** The vmPFC region-of-interest used for all fMRI analyses (following^60^) **b** Full representational-similarity matrix indexing the similarity (expressed as Pearson correlation coefficients) between the activity patterns of any two simulated episodes. **c** To test whether the vmPFC carried information about individual people and places, we examined the replicability of the associated activity patterns across phases I and III. If the vmPFC codes for information about particular people and places, we expect greater similarity for the repetition of the same element (within-item similarity) than for the comparison of an element with a different element of the same category (e.g., other places paired with liked people) (between-item similarity). **d** Consistent with this prediction, we observed greater within-item than between-item similarity across the different categories. The activity pattern in the vmPFC thus carries information about individual, personally known people and places. Boxplots indicate the median, central quartiles, and + /− 2.7 SD. Dots denote outliers beyond that range. pl,  place

We assessed the specific activity pattern associated with each simulation by modeling the fMRI time series with a separate regressor for every episode. For each regressor, we then calculated the *t*-values of the parameter estimates^26^ and extracted a vector of all *t*-values from the voxels within an anatomical mask of the vmPFC (Fig. 2a). A vector thus characterizes the activity pattern for a specific episodic simulation. Next, we quantified the neural similarity of any two simulations by computing the Pearson correlation of their activity patterns, yielding values ranging from 1 (i.e., greatest similarity) to −1 (i.e., greatest dissimilarity). We then analyzed the (Fisher*-z*-transformed) similarity values with a repeated-measures ANOVA that included the factors comparison (within, between), material (people, places), and valence (liked, disliked) (Fig. 2). In addition to a significant effect of material (*F*_1,17 _= 4.94, *p* = 0.04, *η*^*2*^ = 0.23), reflecting overall greater similarity for people than places, we also obtained the critical effect of comparison (*F*_1,17 _= 18.86, *p* < 0.001, *η*^*2*^ = 0.53) (see also Supplementary Fig. 2 for a control analysis corroborating that the greater within-item similarity does not merely reflect less variability of the activity patterns due to less variability in value for an item with itself than with other items).

Episodic simulations are thus associated with replicable activity patterns in vmPFC that are more similar for repeated simulations of the identical element than for any two simulations of different elements. The results support the premise that this region codes for representations that can be re-instated during episodic simulation^16,17^. In the next section, we examine whether activation in the same region-of-interest (ROI) also contains information about associated affect.

### vmPFC activity reflects the value of the simulated element

If vmPFC representations entail the affective value of specific elements, this should be reflected in the activation profile of this region^19^. We performed a parametric-modulation analysis of the BOLD time series using the liking scores as an index of the elements’ respective affective value. Given that the integrative simulation of people and places changed their affective value, we used the liking ratings of the pre-rating for phase I and of the post rating for phase III. (Note that the people contribute more variance in value to the analysis: they included liked and disliked exemplars, whereas the places were selected to be neutral. However, the model also entails the prediction that activation for the neutral people falls in between activation for the liked and disliked people).

**Fig. 3:**
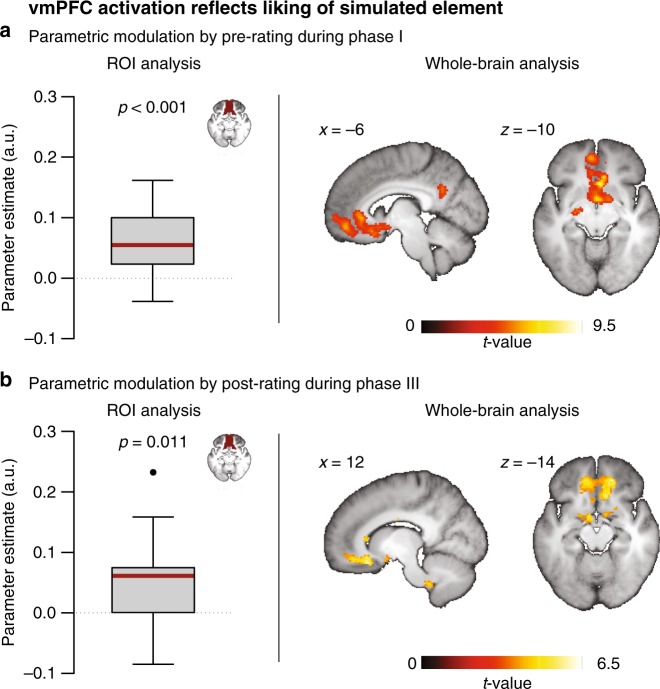
vmPFC activity reflects the value of the imagined element. Consistent across (**a**) phase I and (**b**) phase III, BOLD signal in the vmPFC was modulated by liking (as an index of value) of the people and places. Representations encoded in this region thus seem to carry information about the elements’ affective value. Note that the greatest source of variance in value stems from the inclusion of liked and disliked people, though the model also predicts that BOLD signal for the neutral places falls in between those extremes. Boxplots indicate the median, central quartiles, and + /− 2.7 SD. The dot indicates an outlier beyond that range. For display purposes, exploratory whole-brain maps are thresholded at *p* < 0.001, uncorrected, with a cluster extend of at least 15 voxels. a.u.,  arbitrary units

Mirroring the ROI of the RSA analysis, we averaged across all parameter estimates within the anatomical mask of the vmPFC. Importantly, this analysis demonstrated that, for both time periods, activation in this region was modulated by the value of the simulated event (phase I: *t*_17 _= 4.23, *p* < 0.001, *d* = 1; phase III: *t*_17 _= 2.85, *p* = 0.011, *d* = 0.67) (Fig. 3). The results were further corroborated by complementary whole-brain analyses. These revealed consistent modulation of brain activation in a cluster that included parts of the vmPFC (Fig. 3; Supplementary Table 3).

The foregoing analyses support the hypothesis that the vmPFC codes for affective representations of our environment that are engaged during episodic simulations. In the following, we examine the proposal that a transfer of affective value between such representations mediates the observed changes in attitude.

### vmPFC activity predicts simulation-induced attitude shifts

Our hypothesis posits that simulations change attitudes by transferring affective value from the UCS (i.e., the person) to the CS (i.e., the place). On one hand, during integrative simulations, activation in the vmPFC should thus be modulated by the liking of the UCS, reflecting its affective value. On the other hand, the activation should be predictive of the ensuing change in attitude toward the CS, indicating the transfer of affective value.

**Fig. 4:**
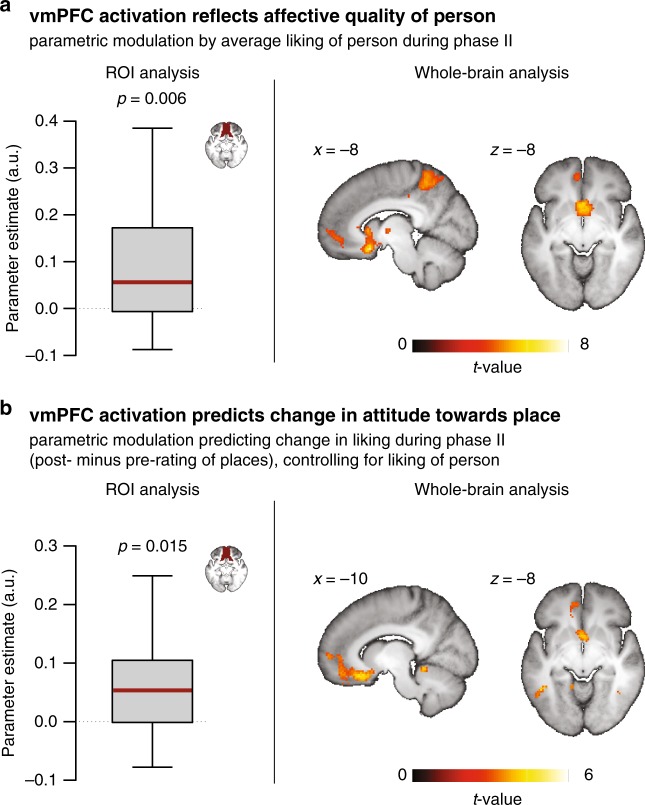
Transfer of affective value from the UCS to the CS during integrative simulations. **a** BOLD signal in the vmPFC was modulated by the liking of the UCS (i.e., the person), reflecting the contribution of its affective value to the simulation. (For display purposed, exploratory whole-brain maps are thresholded at *p* < 0.001, uncorrected with a cluster extend of at least 15 voxels.) **b** Even controlling for (**a**), BOLD signal in the vmPFC further predicted the ensuing change in liking of the CS, thus indicating a transfer of affective value (from the liked UCS to their paired CS). (For display purposes, exploratory whole-brain maps are thresholded at *p* < 0.005, uncorrected with a cluster extend of at least 15 voxels.) Boxplots indicate the median, central quartiles, and + /− 2.7 SD. a.u.,  arbitrary units

To test these two predictions, we performed a parametric-modulation analysis of the fMRI time series obtained during phase II. As a first modulator, we included the liking of the person (averaged across the pre- and post rating). This regressor thus yields regions where activation varies with the affective value of the UCS. As a second modulator, we included the change in liking for the respective place (i.e., post- minus pre-rating). This regressor thus identifies regions where greater activation during the joint simulations predicts a more positive shift in attitude for the CS (even when controlling for linear effects of UCS value). Both regressors yielded the predicted modulations of vmPFC activation in our ROI (liking of UCS: *t*_17 _= 3.16, *p* = 0.006, *d* = 0.75; change in liking of CS: *t*_17 _= 2.7, *p* = 0.015, *d* = 0.64) (Fig. 4). Again, this pattern was also evident in exploratory whole-brain analyses (Fig. 4; Supplementary Table 4).

The predictive signal in the vmPFC was also reliable when we analyzed the ROI data without controlling for the affective value of the UCS (a Shapiro–Wilk test suggested a deviation from normality: *W* = 0.83; *p* = 0.004, thus using a Wilcoxon test: *W*_17_ = 141, *p* = 0.014, matched rank-biserial correlation *r* = 0.65). It was moreover significant when we controlled for the plausibility of the pairing (*t*_17 _= 2.48, *p* = 0.024, *d* = 0.58) (Supplementary Fig. 3).

Critically, given the difference in familiarity for liked versus disliked people, it is important to note that we also obtained this effect when controlling for familiarity of the paired person—either by including it as a first parametric regressor (*t*_17 _= 2.65, *p* = 0.017, *d* = 0.62) or by first regressing out the contribution of familiarity from the individual change scores and then performing the parametric-modulation analysis based on the residuals (using a Wilcoxon test: *W*_17_ = 136, *p* = 0.027, matched rank-biserial correlation *r* = 0.59 due to a significant Shapiro–Wilk test: *W* = 0.88; *p* = 0.029) (Supplementary Fig. 3).

Beyond the vmPFC, previous studies have associated the hippocampus with the transfer of value between arbitrary and novel stimuli^11,28^. We did not observe such a concomitant effect for an anatomical mask of this control region (*t*_17 _= 0.04, *p* = 0.97, *d* = 0.009).

In summary, activation in the vmPFC was modulated by the affective value of the UCS and predicted the subsequent positive change in liking of the CS. The results therefore provide support for the hypothesized transfer of affective value, at least from the liked UCS to their paired CS. At the same time, they provide more general insights into the functions supported by the vmPFC.

## Discussion

It is a long-standing view that the PFC supports control processes that operate on representations stored in posterior brain regions^29^. Somewhat contrary to this ostensible dichotomy, it has been suggested that the medial PFC also creates (schematic) representations of the environment, presumably by extracting commonalities across different episodes^12–14^. However, though activity patterns within this region have been shown to carry information about, for example^29^, individual people^30,31^, locations^32^, or the degree of connectedness within a social network^33^, there is scarce evidence that the vmPFC codes more generally for representations of our environment. The current data provide such evidence: in the vmPFC, replicable activity patterns emerged for both, particular known people and specific familiar places.

Though the current data indicate that the vmPFC represents information about individual entities (i.e., of individual people and places), this observation does not preclude the possibility that the representations are organized in a higher-level structure. Indeed, we further observed an overall greater pattern similarity for people than places, indicating that this region also codes for categorical information. More generally, neuroimaging evidence indicates that the medial PFC acts as a hub that integrates diverse information that is distributed across the brain^15,17,34–36^. The integration may take the form of a dimension reduction that only codes for the information that is currently most relevant^35,37^. Accordingly, the vmPFC may represent information along (hidden) dimensions rather than coding for distinct entities per se.

A dimensional coding is also consistent with previous observations that the relative activation in the vmPFC, when thinking about oneself versus other people, scales with the perceived similarity of the other person to oneself^38,39^. This suggests that the vmPFC does not code for individual people per se but for individual features along continuous dimensions that, in turn, differentiate individual people^34^.

Importantly, in phases I and III, we observed that activation in the vmPFC also reflects the affective value of the constituting elements of an episode, with an increase in activation from disliked via neutral to liked elements. The data are thus consistent with the hypothesis that the vmPFC codes for a continuum of value ranging from negative to positive rather than for other features, such as salience^19,40^.

More specifically, the data support the proposal that representations in the vmPFC integrate conceptual information with associated affect and thus code for “hot“ models of the world^17,18,25^. In phase II of this study, the affective value of an episode was likely determined by the valence of the UCS (i.e., the person), given that the paired CS (i.e., the place) had been selected to be initially neutral. This point was corroborated by the finding that vmPFC activation during the integrative simulations was modulated by the liking of the respective featured person. Behaviorally, it was also reflected in a greater experienced pleasantness for episodes featuring liked than disliked people.

Importantly, the value signal in the vmPFC during episodic simulation has previously been shown to go beyond the value of the individual elements of the episode. That is, even when controlling for the nominal value of the constituting elements, this region signals the anticipated emergent value of the imagined scenario^17^. Episodic simulation may contribute to the processing of such emergent value by emphasizing the elements’ features that are particularly salient in the imagined event^22,23^. It thus affords an estimate of context-specific value that can deviate from the value that is more commonly attached to a given element. Similarly, in this experiment, we observed that vmPFC activation did not just reflect the value of the UCS. It moreover predicted the ensuing shift in attitude toward the CS. We suggest that this vmPFC signal reflects a prediction error regarding the CS that indicates the degree to which the experienced affect deviates from the expectation (e.g., more pleasant than expected; see also refs. ^22,41^). This signal may then drive plasticity in the representation of the CS and lead to the updating of its value^41^.

Given that the vmPFC signal codes for a continuum from negative to positive value^19,40^, this mechanism may generally support both downward and upward value-updating. However, in these experiments, the CS that had been imagined with disliked UCS also showed a positive—albeit weaker—shift in liking (see below). It thus remains to be determined whether this mechanism can also lead to a downward shift via a transfer of negative affective value.

We had hypothesized a particular involvement of the vmPFC in mediating simulation-induced attitude changes due to the region’s dual contribution to the representation of schemas^12–15^ and the processing of value^19^. However, we do not suggest that this region performs this function in isolation. Specifically, the striatum has long been associated with the transfer of value from a UCS to a CS^42^. Our data indicate that striatal activity also tracks the value of the imagined UCS during the simulation of new events (see also ref. ^17^). This information may then be conveyed to the vmPFC and interact with the existing schematic representation of the CS to process an updated value estimate.

It is noteworthy that we did not observe concomitant effects in the hippocampus. This region, and its interactions with the striatum, has previously been implicated in the transfer of value^11,28^. We think it is critical to note that these studies examined such transfer between arbitrary combinations of novel stimuli. The hippocampus may be particularly important in such situations, because they require the rapid encoding of both the individual items and their relations^43,44^, see also ref. ^45^. By contrast, in this study, we examined changes in attitude towards well-established elements from participants’ real-life environment. As such, the integrative simulations could be based on the co-activation of established knowledge structures that are already represented in the vmPFC^12,17^. Therefore, simulation-induced attitude changes may be less reliant on hippocampal processes than more episodic forms of value transfer (as in ref. ^45^). It will be an important avenue for future studies to systematically delineate the contributions of the striatum, hippocampus, and vmPFC as well as their interactions^46,47^.

Episodic simulation has previously been shown to have powerful influences on how we perceive and plan for the future. It increases the perceived plausibility of a prospective scenario^48,49^ and conveys its anticipated affective experience^8,17,21^. This experience, in turn, can foster more farsighted decisions by increasing the salience of future rewards^7^. Similarly, simulations of even unlikely future threats can help avoiding grave danger^50,51^. However, such simulations may also contribute to the development of depression and anxiety^52,53^. The current data show that imaginings can further have a fundamental impact on how we evaluate our environment in the present.

In the fMRI study, we observed that merely imagining meeting a known person at a familiar place can boost the value that we attach to that location. Importantly, we obtained the same effect, with a similar effect size, in a pre-registered study with a larger sample size, thus demonstrating the replicability of the simulation-induced attitude change. The extent of this effect was associated with vmPFC activation during the integrative simulations. This observation indicates that the attitude change was induced at that stage rather than as a process of deliberative revaluation during the post test.

Somewhat surprisingly, we also consistently observed a positive change in liking for places imagined with disliked people. We caution that our design did not include a baseline condition (such as neutral places imagined with neutral people) that would have allowed us to infer simple effects, such as mere exposure^54^, that could potentially account for a general positive shift in liking. Such an effect may boost the value of even those places that had been imagined with disliked people, thus possibly masking any downward impact of simulations. Importantly, however, the change in attitude was more positive for places that had been the location for meetings with liked than with disliked people, indicating a critical influence of the UCS’s valence. The results thus demonstrate how mere imaginings can have a similar impact on our attitudes as real happenings^9,10^.

The observation that episodic simulations can change our attitudes toward the very basic elements that the simulations had been based on has potentially wide-ranging implications. Exaggerated simulations of prospective rewards and threats can provide adaptive benefits by inducing biases that motivate farsighted decisions^50,51^. Critically, however, the current data suggest that exaggerated simulations can also produce a distorted model of the environment that becomes decoupled from actual experiences. This mechanism going awry may thus contribute to the development and maintenance of mental health problems such as depression, bipolar disorder, and anxiety that are often characterized by pronounced prospective thoughts^52,53,55^. More generally, the findings highlight the powerful function of simulations not just in guiding future-oriented decisions but also in creating our models of the world.

## Methods

### Participants

All participants reported no history of psychiatric or neurological disorders and gave informed consent as approved by the Harvard University Institutional Review Board (fMRI study) and the ethics committee of the University of Leipzig (replication study). All thirty participants of the fMRI study were right-handed, native English speakers, who all had normal (or corrected to normal) vision. Twelve participants had to be excluded either because of falling asleep in the scanner (two) or excessive head movements (ten) (defined as maximal absolute motion > 3 or more than five individual movements > 0.5 mm in any functional run). We thus included data from 18 participants (3 male; mean age: 21.33 y; range: 18–27 y). The replication study included 30 native German speakers (17 male; mean age: 23.97 y; range: 20–32 y) (as pre-registered to provide 80% power to detect an effect size of ~2/3 the original; https://aspredicted.org/9ti3h.pdf).

### Tasks and procedure—fMRI study

The procedure, adapted from ref. ^17^, comprised a preparation and a simulation session (Fig. 1a). During the preparation session, participants provided 100 places and 150 people that they were personally familiar with. Of the people, 100 had to be ones that they much liked, 30 people that they felt neutral toward, and 20 people that they much disliked. Participants then rated on nine-point scales (i) how familiar they were with each person and place (1: unfamiliar; 5: intermediate; 9: very familiar), indicating the degree of knowledge, and (2) how much they liked that given item (1: much dislike; 5: neutral; 9: much like), indicating the affective value.

We then selected 28 neutral places (i.e., with a rating of 5 and, if necessary, additional places with the next smaller and greater ratings), the 14 most liked people, and 14 of the least liked people. Piloting indicated that, overall, disliked people tended to be less familiar than liked people. To minimize this gap, we selected the disliked people (of all those that had received a likableness rating smaller than 4, or, if necessary, with the next greater ratings) that were most familiar. (However, the mean Pearson correlation between liking and familiarity across liked and disliked people remained at *r* *=* 0.58, *SD* = 0.31). Finally, we randomly combined each neutral place (i.e., the CS) with either a liked or disliked person (i.e., the UCS), thus creating 14 pairings in each condition.

At the beginning of the simulation session, participants received training on the tasks for the different phases (with items that were not part of the critical pairings). On any trial of phases I and III, they were presented with a fixation cross for 500 ms, followed by a person or a place from the critical pairings for 7.5 s. During this time, participants imagined an episode of interacting with the person or place. They were instructed to imagine the episode as vividly as possible, ensuring that they have a clear mental picture of the respective item. They then rated the vividness of their imagination on a five-point scale within a maximum of 3 s. The remainder of the maximal response time, if any, was added to the subsequent ITI, which lasted for at least 3 s plus an additional jittered period (0–8 s in 2 -s intervals). The screen during the ITI was blank. In phase II, participants were presented with both the person and place of a given pairing, and then imagined an interaction with the person that would be specific to the given place as in ref. ^17^.

The MRI session began with a resting-state scan (not reported), before participants entered phase I. Here, they imagined each person and place across two functional runs. The person and place of a given pairing appeared in the same run in a pseudorandom order, with the constraint that one item appeared in the first and the other in the second half. During phase II, participants encountered each pairing three times in as many functional runs (pairs were presented in a different random order for each run). Participants were instructed to keep imagining the same episode for a given pairing, adding in more details and attempting to make it as vivid as possible. We had chosen three repetitions, because piloting indicated (i) that this was not too strenuous for the participants and (ii) that it was sufficient to yield the behavioral effect. Phase III repeated phase I, though with a newly pseudo-randomized presentation order and the additional constraint that the items that had been presented in the first run of phase I were also presented in the first run of phase III. Following this phase, participants performed a localizer task as in ref. ^17^ (results not reported).

Outside the scanner, we assessed the main behavioral-dependent measure: Participants were shown each person and place in a random order and indicated their respective liking on the same scale as in the preparation session. Finally, participants were shown each pairing in a random order and rated the plausibility of such a meeting and its anticipated pleasantness (both on nine-point scales).

### Tasks and procedure—replication study

The overall procedure of the pre-registered replication (https://aspredicted.org/9ti3h.pdf) was identical to the fMRI study, except for the omission of phases I and II. Moreover, to match the liked and disliked people in terms of familiarity, we used an alternative selection approach (Supplementary Methods).

### fMRI acquisition

Using a 3 Tesla Siemens Magnetom TimTrio MRI scanner with a 32-channel head coil, we acquired anatomical images with a T1-weighted magnetization-prepared rapid gradient multi-echo sequence (MEMPRAGE, 176 sagittal slices, TR = 2530 ms, TEs = 1.64, 3.50, 5.36, and 7.22 ms, flip angle = 7°, 1 mm^3^ voxels, FoV = 256 mm). During each of seven functional runs, we acquired 220 volumes of blood-oxygen-level-dependent (BOLD) data with a T2*-weighted echo-planar imaging (EPI) pulse sequence that employed multiband RF pulses and Simultaneous Multi-Slice (SMS) acquisition^56,57^ with the following parameters: 69 interleaved axial–oblique slices (angled 17° toward coronal from ACPC), TR = 2000 ms, TE = 27 ms, flip angle = 80°, 2 × 2 × 2 mm^3^ nominal voxels, 6/8 partial fourier, FoV = 216 mm, SMS = 3. The first five volumes of each run were discarded to allow for T1 equilibration effects.

### fMRI analysis

Data were analyzed using SPM12 (www.fil.ion.ucl.ac.uk/spm). The functional images were realigned, corrected for slice acquisition times, and coregistered with the structural image. This was spatially normalized and the resulting parameters served to normalize the functional images by fourth-degree B-spline interpolation (preserving the functional voxel resolution of 2 × 2 × 2 mm^3^ isotropic) to the Montreal Neurological Institute reference brain. The images were then smoothed by an isotropic 8 mm full-width half-maximum Gaussian kernel for the general linear models (GLM) assessing parametric modulations. The GLM that provided the input for RSA was based on unsmoothed data.

The GLMs analyzed regional activity by decomposing the variance in the BOLD time series, separately for each functional run^58^. Each model included six regressors representing residual movement artifacts and the mean over scans. A further regressor coded for the onsets and durations of trials for which participants did not provide a rating in time, if applicable. The additional regressors in a given GLM coded for the respective effects-of-interest by analyzing the remaining trials.

A first GLM assessed brain activation associated with the affective value of simulated items during phases I and III. We therefore entered a regressor coding for the duration of all simulation trials plus an additional parametric regressor coding for the liking of the respective simulated item. Given that the paired simulations in phase II changed attitudes, we used the pre-ratings for phase I and post ratings for phase III.

A second GLM assessed brain activation associated with the transfer of affective value during the integrative simulations in phase II. We entered (i) a regressor coding for the duration of all simulations, (ii) a first parametric modulator coding for the affective value of the UCS (i.e., liking of the person, averaged across pre- and post rating), and (iii) a second parametric modulator coding for the change in value of the CS (i.e., post- minus pre-rating liking of the place). The first parametric regressor reveals regions where activation is modulated by the value of the UCS, whereas the second regressor indicates where the residual activation is greater in case of a more positive change in liking of the CS. Additional GLMs corroborated effects of affect transfer without controlling for the effect of the UCS and controlling for the plausibility of the pairing. In two additional analyses, we further established this effect by controlling for the familiarity of the UCS—either by including it as a first parametric regressor or by computing the analysis based on the residuals of the change scores after regressing out possible effects of familiarity.

A final GLM estimated activity patterns separately for each simulation during phases 1 and 3 (thus including 112 regressors, one for each of the two simulations of the 28 places and 28 people). The ensuing parameters were used for the RSA^26,59^ that tested for individual representations in vmPFC.

All trial regressors were convolved with the canonical hemodynamic response function. A 1/128-Hz high-pass filter was applied to the data and the respective model, and parameter estimates for each regressor were calculated from the least-mean-squares fit of the model to the data.

Following Liu, Grady, and Moscovitch^60^, an anatomical mask of our region-of-interest, the vmPFC, was created by merging the gyrus rectus and the medio-orbital section of the frontal gyrus of the AAL template^61^ using the WFU-Pickatlas toolbox^62^ (Fig. 2a). For univariate effects, we extracted parameter estimates, for each participant, from this a priori ROI. For complementary and exploratory whole-brain analyses, the respective contrast estimates were entered into a second-level analysis, where we used cluster-level inference at *p* < 0.05 (FWE-corrected) with a cluster forming threshold of *p* < 0.001 and at least 15 contiguous voxels. These analyses also employed the vmPFC mask for targeted small-volume-correction. In addition, for an exploratory analysis, we also used the AAL template^61^ to create a bilateral mask of the hippocampus.

The RSA analyses were conducted using the toolbox by Nili et al.^59^. We only included trials on which participants had provided a response within the allotted time. Analyses were based on the *t*-values of the estimated parameter estimates from each voxel within our ROI. Within-item similarity was assessed, for each person and place, by computing the Pearson correlation of these values between phases 1 and 3. Between-item similarity was only based on the correlations between elements of the same material and valence (e.g., only between liked people), to ensure that the results are not driven by category differences in neural coding. Moreover, due to temporal autocorrelations of noise, the activity patterns of proximal events tend to be more similar than of those events that are more distant in time^63^. To quantify between-item similarity, we therefore only included similarity values of the same functional run as for the corresponding within-item comparison (i.e., correlating events from the 1st and 6th as well as from the 2nd and 7th functional runs only). Inferential statistics were based on Fisher*-z*-transformed correlation values.

### Reporting summary

Further information on research design is available in the Nature Research Reporting Summary linked to this article.

## Supplementary information

Supplementary Information

Peer Review

Reporting Summary

## Data Availability

The data that support the findings of this study are available from the corresponding author upon reasonable request.

## Code availability

The code used for the analyses is available from the corresponding author upon request.

## References

[1] Schacter DL, Benoit RG, Szpunar KK (2017). Episodic future thinking: mechanisms and functions. Curr. Opin. Behav. Sci..

[2] Suddendorf T, Corballis MC (2007). The evolution of foresight: what is mental time travel, and is it unique to humans?. Behav. Brain Sci..

[3] Benoit RG, Schacter DL (2015). Specifying the core network supporting episodic simulation and episodic memory by activation likelihood estimation. Neuropsychologia.

[4] Hassabis D, Kumaran D, Maguire EA (2007). Using imagination to understand the neural basis of episodic memory. J. Neurosci. J. Soc. Neurosci..

[5] Irish M, Addis DR, Hodges JR, Piguet O (2012). Considering the role of semantic memory in episodic future thinking: evidence from semantic dementia. Brain.

[6] Schacter DL, Addis DR (2007). The cognitive neuroscience of constructive memory: remembering the past and imagining the future. Philos. Trans. R. Soc. B Biol. Sci..

[7] Benoit, R. G., Berkers, R. M. W. J. & Paulus, P. C. An adaptive function of mental time travel: motivating farsighted decisions. *Behav*. *Brain Sci.* **41**, e3 (2018). 10.1017/S0140525X1700125X 29353564

[8] Demblon J, D’Argembeau A (2016). Networks of prospective thoughts: the organisational role of emotion and its impact on well-being. Cogn. Emot..

[9] Hofmann W, De Houwer J, Perugini M, Baeyens F, Crombez G (2010). Evaluative conditioning in humans: a meta-analysis. Psychol. Bull..

[10] Jones CR, Olson MA, Fazio RH (2010). Evaluative conditioning: the ‘how’ question. Adv. Exp. Soc. Psychol..

[11] Wimmer GE, Shohamy D (2012). Preference by association: how memory mechanisms in the hippocampus bias decisions. Science.

[12] Milivojevic B, Vicente-Grabovetsky A, Doeller CF (2015). Insight reconfigures hippocampal-prefrontal memories. Curr. Biol..

[13] Richter FR, Chanales AJH, Kuhl BA (2016). Predicting the integration of overlapping memories by decoding mnemonic processing states during learning. NeuroImage.

[14] Schlichting ML, Mumford JA, Preston AR (2015). Learning-related representational changes reveal dissociable integration and separation signatures in the hippocampus and prefrontal cortex. Nat. Commun..

[15] Gilboa A, Marlatte H (2017). Neurobiology of schemas and schema-mediated memory. Trends Cogn. Sci..

[16] Barron HC, Dolan RJ, Behrens TEJ (2013). Online evaluation of novel choices by simultaneous representation of multiple memories. Nat. Neurosci..

[17] Benoit RG, Szpunar KK, Schacter DL (2014). Ventromedial prefrontal cortex supports affective future simulation by integrating distributed knowledge. Proc. Natl. Acad. Sci..

[18] Metcalfe J, Mischel W (1999). A hot/cool-system analysis of delay of gratification: dynamics of willpower. Psychol. Rev..

[19] Bartra O, McGuire JT, Kable JW (2013). The valuation system: a coordinate-based meta-analysis of BOLD fMRI experiments examining neural correlates of subjective value. NeuroImage.

[20] Peters J, Büchel C (2010). Neural representations of subjective reward value. Behav. Brain Res..

[21] Benoit RG, Gilbert SJ, Burgess PW (2011). A neural mechanism mediating the impact of episodic prospection on farsighted decisions. J. Neurosci..

[22] Lin WJ, Horner AJ, Bisby JA, Burgess N (2015). Medial prefrontal cortex: adding value to imagined scenarios. J. Cogn. Neurosci..

[23] Lin WJ, Horner AJ, Burgess N (2016). Ventromedial prefrontal cortex, adding value to autobiographical memories. Sci. Rep..

[24] Shenhav A, Barrett LF, Bar M (2013). Affective value and associative processing share a cortical substrate. Cogn. Affect. Behav. Neurosci..

[25] Roy M, Shohamy D, Wager TD (2012). Ventromedial prefrontal-subcortical systems and the generation of affective meaning. Trends Cogn. Sci..

[26] Kriegeskorte, N., Mur, M. & Bandettini, P. Representational similarity analysis – connecting the branches of systems neuroscience. *Front. Syst.**Neurosci*. **2**, 2-4 (2008). 10.3389/neuro.06.004.2008 PMC2605405 19104670

[27] Charest I, Kievit RA, Schmitz TW, Deca D, Kriegeskorte N (2014). Unique semantic space in the brain of each beholder predicts perceived similarity. Proc. Natl. Acad. Sci..

[28] Gilboa A, Sekeres M, Moscovitch M, Winocur G (2014). Higher-order conditioning is impaired by hippocampal lesions. Curr. Biol. CB.

[29] Miller EK, Cohen JD (2001). An integrative theory of prefrontal cortex function. Annu. Rev. Neurosci..

[30] Szpunar KK, Jacques PLS, Robbins CA, Wig GS, Schacter DL (2014). Repetition-related reductions in neural activity reveal component processes of mental simulation. Soc. Cogn. Affect. Neurosci..

[31] Thornton MA, Mitchell JP (2018). Theories of person perception predict patterns of neural activity during mentalizing. Cereb. Cortex.

[32] Robin J, Buchsbaum BR, Moscovitch M (2018). The primacy of spatial context in the neural representation of events. J. Neurosci..

[33] Parkinson C, Kleinbaum AM, Wheatley T (2017). Spontaneous neural encoding of social network position. Nat. Hum. Behav..

[34] Hassabis D (2014). Imagine all the people: how the brain creates and uses personality models to predict behavior. Cereb. Cortex.

[35] Lim SL, O’Doherty JP, Rangel A (2013). Stimulus value signals in ventromedial PFC reflect the integration of attribute value signals computed in fusiform gyrus and posterior superior temporal gyrus. J. Neurosci..

[36] van Kesteren MTR, Ruiter DJ, Fernández G, Henson RN (2012). How schema and novelty augment memory formation. Trends Neurosci..

[37] Mack, M. L., Preston, A. R. & Love, B. C. Medial prefrontal cortex compresses concept representations through learning. Preprint at https://www.biorxiv.org/content/10.1101/178145v1 (2017).

[38] Benoit RG, Gilbert SJ, Volle E, Burgess PW (2010). When I think about me and simulate you: medial rostral prefrontal cortex and self-referential processes. NeuroImage.

[39] Mitchell JP, Macrae CN, Banaji MR (2004). Encoding-specific effects of social cognition on the neural correlates of subsequent memory. J. Neurosci..

[40] Litt A, Plassmann H, Shiv B, Rangel A (2011). Dissociating valuation and saliency signals during decision-making. Cereb. Cortex.

[41] Garrison J, Erdeniz B, Done J (2013). Prediction error in reinforcement learning: a meta-analysis of neuroimaging studies. Neurosci. Biobehav. Rev..

[42] Shohamy D (2011). Learning and motivation in the human striatum. Curr. Opin. Neurobiol..

[43] Eichenbaum H, Cohen NJ (2014). Can we reconcile the declarative memory and spatial navigation views on hippocampal function?. Neuron.

[44] Kumaran D, Hassabis D, McClelland JL (2016). What learning systems do intelligent agents need? Complementary learning systems theory updated. Trends Cogn. Sci..

[45] Wimmer GE, Daw ND, Shohamy D (2012). Generalization of value in reinforcement learning by humans. Eur. J. Neurosci..

[46] Shohamy D, Daw ND (2015). Integrating memories to guide decisions. Curr. Opin. Behav. Sci..

[47] Gerraty RT, Davidow JY, Wimmer GE, Kahn I, Shohamy D (2014). Transfer of learning relates to intrinsic connectivity between hippocampus, ventromedial prefrontal cortex, and large-scale networks. J. Neurosci..

[48] Gregory WL, Cialdini RB, Carpenter KM (1982). Self-relevant scenarios as mediators of likelihood estimates and compliance: does imagining make it so?. J. Pers. Soc. Psychol..

[49] Szpunar KK, Schacter DL (2013). Get real: effects of repeated simulation and emotion on the perceived plausibility of future experiences. J. Exp. Psychol. Gen..

[50] Bulley A, Henry JD, Suddendorf T (2017). Thinking about threats: memory and prospection in human threat management. Conscious. Cogn..

[51] Miloyan B, Suddendorf T (2015). Feelings of the future. Trends Cogn. Sci..

[52] Miloyan B, Pachana NA, Suddendorf T (2014). The future is here: a review of foresight systems in anxiety and depression. Cogn. Emot..

[53] Holmes EA (2011). Mood stability versus mood instability in bipolar disorder: a possible role for emotional mental imagery. Behav. Res. Ther..

[54] Zajonc RB (2001). Mere exposure: a gateway to the subliminal. Curr. Dir. Psychol. Sci..

[55] Benoit RG, Davies DJ, Anderson MC (2016). Reducing future fears by suppressing the brain mechanisms underlying episodic simulation. Proc. Natl. Acad. Sci..

[56] Moeller S (2010). Multiband multislice GE-EPI at 7 tesla, with 16-fold acceleration using partial parallel imaging with application to high spatial and temporal whole-brain fMRI. Magn. Reson. Med..

[57] Setsompop K (2012). Blipped-controlled aliasing in parallel imaging (blipped-CAIPI) for simultaneous multi-slice EPI with reduced g-factor penalty. Magn. Reson. Med..

[58] Friston KJ (1995). Analysis of fMRI time-series revisited. NeuroImage.

[59] Nili H (2014). A toolbox for representational similarity analysis. PLoS Comput. Biol..

[60] Liu ZX, Grady C, Moscovitch M (2017). Effects of prior-knowledge on brain activation and connectivity during associative memory encoding. Cereb. Cortex.

[61] Tzourio-Mazoyer N (2002). Automated anatomical labeling of activations in SPM using a macroscopic anatomical parcellation of the MNI MRI single-subject brain. NeuroImage.

[62] Maldjian JA, Laurienti PJ, Kraft RA, Burdette JH (2003). An automated method for neuroanatomic and cytoarchitectonic atlas-based interrogation of fMRI data sets. NeuroImage.

[63] Alink, A., Walther, A., Krugliak, A., Bosch, J. J. F. van den & Kriegeskorte, N. Mind the drift - improving sensitivity to fMRI pattern information by accounting for temporal pattern drift. Preprint at https://www.biorxiv.org/content/10.1101/032391v2 (2015).

