# Supplementary material for: Forming attitudes via neural activity supporting affective episodic simulations
Source: Nat Commun. 2019 May 17;10:2215. doi: 10.1038/s41467-019-09961-w (PMC6525197; doi:10.1038/s41467-019-09961-w)
Supplement: Supplementary file 1 — Supplementary Information [file 41467_2019_9961_MOESM1_ESM.pdf]

Forming attitudes via neural activity supporting affective episodic simulations

Roland G. Benoit, Philipp C. Paulus, & Daniel L. Schacter

Supplementary Information

Supplementary methods

Matching liked and disliked people on familiarity for the replication study

The overall procedure for the replication study was identical to the main study albeit for the omission of phases I and III. Participants provided names of 100 places and of 150 people that they were personally familiar with. We then asked participants to rate on a 9-point scale (i) how familiar they were with each person and place, and (ii) how much they liked each individual item. We then selected 28 neutral places (i.e., rating of 5 and, if necessary, additional items with the next lower and greater ratings).

Based on the liking and familiarity ratings, we selected two sets of people (i.e., the liked and disliked) such that we (i) maximized the difference in liking and (ii) minimized the difference in familiarity between the sets. To this end, we used a stepwise selection approach. First, we used linear regression to remove shared variance of familiarity and liking from the raw liking scores. We then selected the 14 people with highest and the 14 people with lowest residual liking scores. We then checked whether the two sets were of equal average familiarity. If this was not the case, we sought to match the two sets by replacing people from the set with the lower average familiarity. That is, of the least familiar people we took either the most liked person (for the disliked set) or the least liked person (for the liked set) and exchanged it with the respective next 'best' person (i.e., the most familiar person with a liking rating smaller than four or greater than 6, respectively). This person was included in the set if it increased the mean familiarity of the set. This approach continued until the sets were matched on familiarity or when the difference could no further be minimized. Finally, we checked if we could include other people of identical familiarity that would further maximize the difference in liking between both sets. We then randomly paired each of the selected liked and disliked people with a unique neutral place to create the critical 28 pairings.

Supplementary note

Detailed results of the replication study

As in the fMRI study, we selected places that participants felt neutral towards and paired these with either much liked or much disliked people (difference in liking: $t_{29} = 23.06$, $p < 0.001$, $d = 4.21$). Importantly, this time, the liked people were not more familiar than the disliked people ($t_{29} = 1.21$, $p = 0.238$, $d = 0.22$) (Supplementary Table 2). (In fact, they were exactly matched for 28 of the 30 participants). The places in the two conditions did also not differ on this dimension (Shapiro-Wilk: $W = 9.26$, $p = 0.038$, hence Wilcoxon: $W_{29} = 143.5$, $p = 0.11$, matched rank biserial correlation $r = -0.38$).

Of the simulated episodes, participants judged those featuring liked people as more plausible ($t_{29} = 4.18$, $p < 0.001$, $d = 0.76$) and, importantly, also as more pleasant ($t_{29} = 19.02$, $p < 0.001$, $d = 3.47$).

We replicated the observation that both kinds of places were deemed more positive following simulation. In the pre-registration, we had specified that we would use *t*-tests, which were indeed significant (paired with liked people: $t_{29} = 6.24$, $p < 0.001$, $d = 1.14$; paired with disliked people: $t_{29} = 3.47$, $p = 0.002$, $d = 0.63$). However, for the change scores of the places paired with liked people, a Shapiro-Wilk test indicated a deviation from normality ($W = 0.93$, $p = 0.046$). We therefore additionally analyzed these data with a Wilcoxon test, which also yielded a significant effect ($W = 439.5$, $p < 0.001$, matched rank biserial correlation $r = 0.89$).

Importantly, as predicted (https://aspredicted.org/9ti3h.pdf), we also replicated the critical finding of a more positive shift in attitude for places that had been imagined with liked people ($t_{29} = 3.77$, $p < 0.001$, $d = 0.69$) (Fig. S1b).

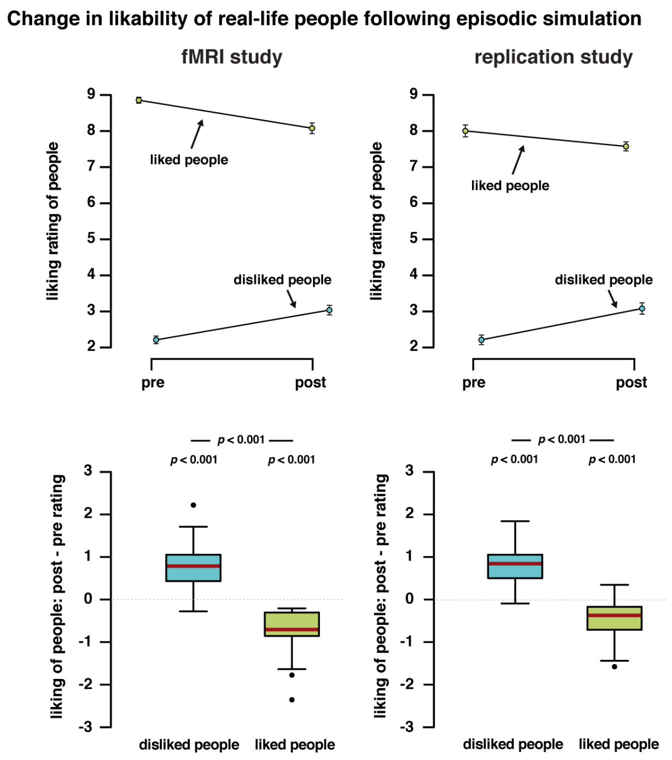

Supplementary Figure 1. Though the studies were designed to examine changes in the liking of the neutral places, we also explored concomitant changes in the liking of the paired liked and disliked people. Consistent across the two studies, the liking of the liked people decreased, whereas the liking of the disliked people increased from the pre- to the post test (with significant differences between the two respective difference scores) (*study 1*: *liked people*: Wilcoxon test: $W_{17} = 0$, $p < 0.001$, matched rank serial correlation $r = -1$, because of significant Shapiro-Wilk: $W = 0.83$, $p = 0.004$; *disliked people*: $t_{17} = 5.98$, $p < 0.001$, $d = 1.41$; *difference:* Wilcoxon test: $W_{17} = 0$, $p < 0.001$, matched rank serial correlation $r = -1$, because of significant Shapiro-Wilk: $W = 0.87$, $p = 0.021$) (*study 2*: *liked people*: $t_{29} = -5.16$, $p < 0.001$, $d = -0.94$; *disliked people*: $t_{29} = 10.7$, $p < 0.001$, $d = 1.95$; *difference:* $t_{29} = -11.08$, $p < 0.001$, $d = -2.02$). Though this pattern is consistent with the hypothesized transfer of value between the constituting elements of a simulation (i.e., the valenced person and the neutral place), we caution any interpretation. The studies were not designed to include a proper baseline to evaluate the changes for the people, and we therefore cannot rule out simple explanations such as regression to the mean (i.e., from either very positive or very negative to the neutral "mean"). Error bars in the pre- vs. post panels indicate the respective standard error of the means. Boxplots indicate the median, central quartiles, and +/- 2.7 SD. The dots indicate outliers beyond that range.

Replicability analysis: within-item vs. matched-liking similarity

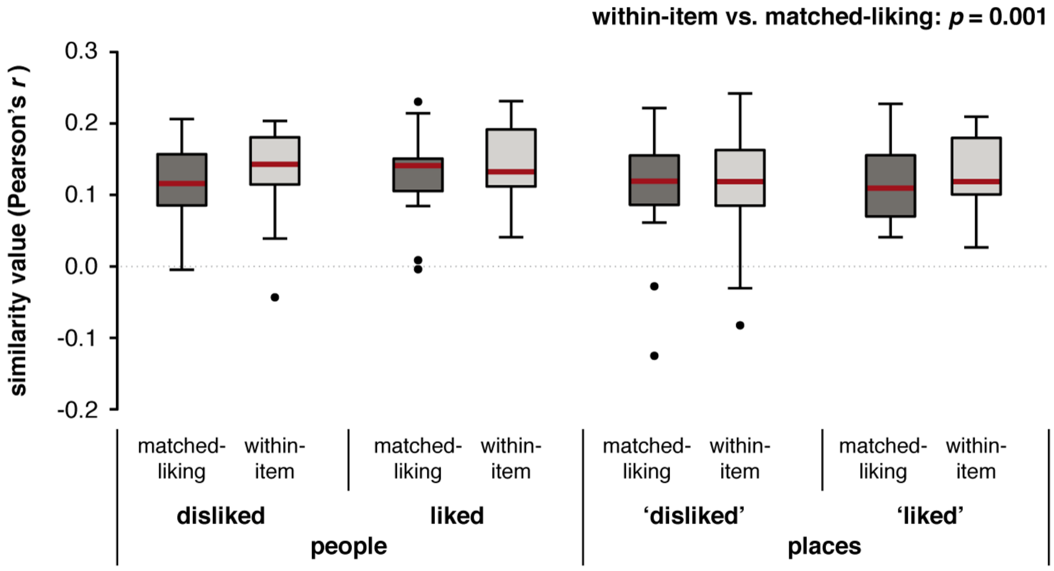

Supplementary Figure 2. Our fMRI results indicate that univariate vmPFC activity is sensitive to value (Fig. 2 & 3)[see also 1,2]. This univariate effect, in turn, may drive differences between multivariate activity patterns for items that differ in value. The current analysis examines whether we can observe evidence for unique representations for individual people and places, even if we compare their within-item similarity not broadly to all other items of the same category (as we had done with the between-item similarity). Instead, we compare the within-item similarity to the similarity of items that are exactly matched in terms of value (i.e., matched-liking similarity). We therefore attempted to pair each item with another item (of the same category and from the same functional run) that had received the identical liking rating on the post test. We thus ensure that the comparison of the item with itself (within-item similarity) versus with its paired item (matched-liking similarity) is not biased by possible value differences. This analysis is based on 78.08 % of the items for which it was possible to assign a matched 'partner'. For many items, there was more than one possible match. Therefore, on each of 1000 iterations, we randomly drew one of the possible matches as a partner before then computing the similarity between the items and their respective partners. We finally averaged these similarity scores across all iterations, which we took as an estimate of the matched-liking similarity. Critically, this approach yielded the predicted larger within-item than matched-liking similarity ($F_{1,17}$ = 14.47, p = 0.001, η^2 = 0.46), i.e., a greater within-item similarity even when we had directly controlled for effects of value. The control analysis thus further demonstrates that the vmPFC codes for individual elements from our environment. In addition, only the main effect of material (people vs. places, $F_{1,17}$ = 6.63, p = 0.02, η^2 = 0.28) but no interactions including the comparison factor (i.e., no interaction with within-item vs. matched-item, all $F_{1,17}$ < 0.51, all p > 0.48, all η^2 < 0.03) were significant. Boxplots indicate the median, central quartiles, and +/- 2.7 SD. Dots denote outliers beyond that range.

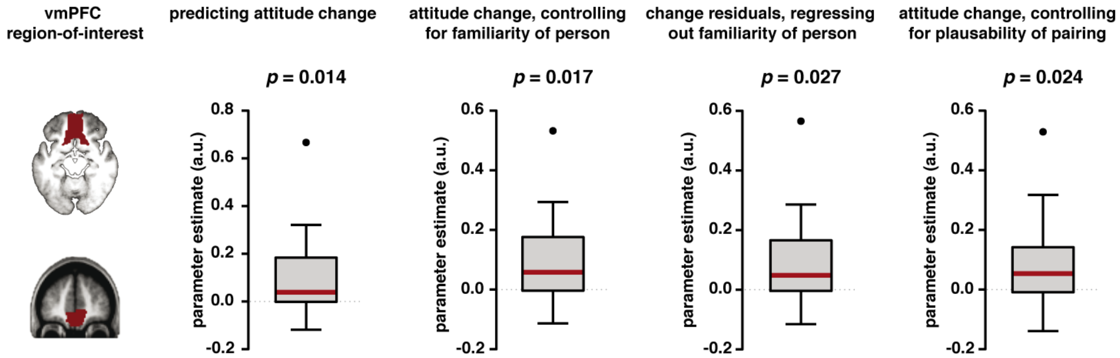

Supplementary Figure 3. Average contrast estimates from the vmPFC region-of-interest indicating that BOLD signal in this region was modulated by the subsequent change in liking of the CS (i.e., the place). This was also the case when controlling for the familiarity of the paired UCS (i.e., the person), either by including this effect as a first parametric regressor or by using the residual change values after regressing out possible effects of familiarity. Moreover, the effect was also present when controlling for the plausibility of the CS-UCS pairing. Boxplots indicate the median, central quartiles, and +/- 2.7 SD. The dots denote an outlier beyond that range.

Supplementary Table 1. Behavioral data of the fMRI study

	Familiarity of people		Familiarity of places		Plausibility		Pleasantness	
	liked	disliked	'liked'	'disliked'	liked	disliked	liked	disliked
**Mean**	7.5	5.3	5.6	5.7	4.6	3.6	7.8	3.2
**Std. Deviation**	0.8	1.5	1.0	0.8	1.0	1.2	0.7	0.8
**Minimum**	5.8	3.2	3.1	4.1	3.3	1.6	6.2	1.6
**Maximum**	8.4	8.4	7.6	7.0	6.6	5.9	8.9	4.3

Supplementary Table 2. Behavioral data of the replication study

	Familiarity of people		Familiarity of places		Plausibility		Pleasantness	
	liked	disliked	'liked'	'disliked'	liked	disliked	liked	disliked
**Mean**	5.5	5.5	6.5	6.7	4.6	3.6	7.7	3.1
**Std. Deviation**	1.2	1.2	1.0	0.9	1.1	1.1	0.8	0.8
**Minimum**	3.4	3.4	4.4	5.0	2.7	2.1	5.3	1.9
**Maximum**	8.3	8.3	8.0	8.5	6.2	5.9	8.9	5.1

Supplementary Table 3. Parametric modulation by affective value (i.e., liking)

Region	approx. BA	Hemisphere	MNI (peak)			Voxels	Z(max)
			x	*y*	*z*		
positive modulation by liking							
vmPFC	11	L/R	6	26	-14	2477	4.93
			6	0	-10	same cluster	4.79
			-10	2	-12	same cluster	4.77
vmPFC	10, 11, 25	L/R	6	26	-14	991*	4.93
			4	24	-10	same cluster	4.74
			-4	20	-16	same cluster	4.24
			-10	38	-16	same cluster	4.02
			-6	26	-12	same cluster	3.99
			-12	40	-10	same cluster	3.84
			4	42	-16	same cluster	3.66
			10	44	-12	same cluster	3.63
			6	16	-20	same cluster	3.61
			12	40	-14	same cluster	3.61
			10	48	-10	same cluster	3.44
			12	50	-4	same cluster	3.32
negative modulation by liking							
vPC/dPC	1, 39	R	36	-30	32	921	3.96
			38	-22	36	same cluster	3.68
			46	-58	46	same cluster	3.55
vPC	7, 39, 40	L	-32	-46	38	459	3.70
			-38	-48	44	same cluster	3.55
			-30	-64	42	same cluster	3.50

Note. Thresholded at $p < 0.05$ FWE-cluster corrected with a cluster forming threshold of $p < 0.001$ and at least 15 contiguous voxels. vmPFC = ventromedial prefrontal cortex, dPC = dorsal parietal cortex, vPC = ventral parietal cortex; * = significant following small-volume-correction for the vmPFC region-of-interest.

We provide coordinates of individual peaks to better characterize the extend of the significant clusters. However, because the results were obtained using cluster-correction, one should not infer that all of the individual peaks are necessarily significantly activated by themselves[3].

Supplementary Table 4. Parametric modulation by the value (i.e., liking) of the UCS (i.e., the person) and by the change in value for the CS (i.e., the place)

Region	approx. BA	Hemisphere	MNI (peak) *x*	*y*	*z*	Voxels	Z(max)
positive modulation by liking of the person							
dlPFC	8	L	-22	26	38	235	3.71
			-20	38	46	same cluster	3.37
vmPFC; CN	25	L/R	-6	14	-18	856	4.95
			8	10	-14	same cluster	4.49
			10	26	6	same cluster	4.22
Precuneus	7	L/R	14	-50	52	648	4.08
			-6	-50	54	same cluster	4.07
			-8	-52	64	same cluster	3.74
dOC	19, 39	L	-44	-76	30	262	4.05
			-40	-70	26	same cluster	3.96
			-36	-84	24	same cluster	3.77
vmPFC	10, 11	L	-6	52	-6	90*	3.46
			-6	44	-14	same cluster	3.17
vmPFC	11, 25	L	-6	14	-20	85*	4.76
			-10	16	-16	same cluster	4.05
			-2	26	-20	same cluster	3.16
negative modulation by liking of the person							
none							
positive modulation by subsequent change in liking of the place							
vmPFC	11	L	-6	16	-22	102*	4.19
			-8	22	-20	same cluster	3.86
			-10	26	-20	same cluster	3.70
			-12	30	-20	same cluster	3.52
			-12	32	-16	same cluster	3.23
negative modulation by subsequent change in liking of the place							
none							

Note. Thresholded at $p < 0.05$ FWE-cluster corrected with a cluster forming threshold of $p < 0.001$ and at least 15 contiguous voxels. vmPFC = ventromedial prefrontal cortex, dlPFC = dorsolateral prefrontal cortex, CN = Caudate Nucleus, dOC = dorsal occipital cortex; * = significant following small-volume-correction for the vmPFC region-of-interest.

We provide coordinates of individual peaks to better characterize the extend of the significant clusters. However, because the results were obtained using cluster-correction, one should not infer that all of the individual peaks are necessarily significantly activated by themselves[3].

Supplementary references

1. Bartra, O., McGuire, J. T. & Kable, J. W. The valuation system: a coordinate-based meta-analysis of BOLD fMRI experiments examining neural correlates of subjective value. *NeuroImage* **76**, 412–427 (2013).
2. Litt, A., Plassmann, H., Shiv, B. & Rangel, A. Dissociating valuation and saliency signals during decision-making. *Cereb. Cortex* **21**, 95–102 (2011).
3. Woo, C.-W., Krishnan, A. & Wager, T. D. Cluster-extent based thresholding in fMRI analyses: pitfalls and recommendations. *NeuroImage* **91**, 412–419 (2014).
