# Supplementary material for: Forming attitudes via neural activity supporting affective episodic simulations
Source: Nat Commun. 2019 May 17;10:2215. doi: 10.1038/s41467-019-09961-w (PMC6525197; doi:10.1038/s41467-019-09961-w)
Supplement: Supplementary file 3 — Reporting Summary [file 41467_2019_9961_MOESM3_ESM.pdf]

nature research

Corresponding author(s): Roland G. Benoit

Reporting Summary

Nature Research wishes to improve the reproducibility of the work that we publish. This form provides structure for consistency and transparency in reporting. For further information on Nature Research policies, see Authors & Referees and the Editorial Policy Checklist.

Statistical parameters

When statistical analyses are reported, confirm that the following items are present in the relevant location (e.g. figure legend, table legend, main text, or Methods section).

n/a	Confirmed	
☐	☒	The exact sample size (n) for each experimental group/condition, given as a discrete number and unit of measurement
☐	☒	An indication of whether measurements were taken from distinct samples or whether the same sample was measured repeatedly
☐	☒	The statistical test(s) used AND whether they are one- or two-sided<br>*Only common tests should be described solely by name; describe more complex techniques in the Methods section.*
☐	☒	A description of all covariates tested
☐	☒	A description of any assumptions or corrections, such as tests of normality and adjustment for multiple comparisons
☐	☒	A full description of the statistics including central tendency (e.g. means) or other basic estimates (e.g. regression coefficient) AND variation (e.g. standard deviation) or associated estimates of uncertainty (e.g. confidence intervals)
☐	☒	For null hypothesis testing, the test statistic (e.g. F, t, r) with confidence intervals, effect sizes, degrees of freedom and P value noted<br>*Give P values as exact values whenever suitable.*
☒	☐	For Bayesian analysis, information on the choice of priors and Markov chain Monte Carlo settings
☐	☒	For hierarchical and complex designs, identification of the appropriate level for tests and full reporting of outcomes
☐	☒	Estimates of effect sizes (e.g. Cohen's d, Pearson's r), indicating how they were calculated
☐	☒	Clearly defined error bars<br>*State explicitly what error bars represent (e.g. SD, SE, CI)*

Our web collection on statistics for biologists may be useful.

Software and code

Policy information about availability of computer code

Data collection	Data were collected using E-Prime 2.0 (https://pstnet.com/products/e-prime/) for the fMRI study and Psychophysics Toolbox (version 3) in Matlab 2017a for the replication study.
Data analysis	Data were analyzed using Matlab 2017a, Matlab 2012a, SPM12 (www.fil.ion.ucl.ac.uk/spm), RSA toolbox (Nili et al., PLoS Comput. Biol., 2014), and JASP (version 0.8, www.jasp-stats.org).

For manuscripts utilizing custom algorithms or software that are central to the research but not yet described in published literature, software must be made available to editors/reviewers upon request. We strongly encourage code deposition in a community repository (e.g. GitHub). See the Nature Research guidelines for submitting code & software for further information.

Data

Policy information about availability of data

All manuscripts must include a data availability statement. This statement should provide the following information, where applicable:

- Accession codes, unique identifiers, or web links for publicly available datasets
- A list of figures that have associated raw data
- A description of any restrictions on data availability

The data that support the findings of this study are available from the corresponding author upon reasonable request.

Field-specific reporting

Please select the best fit for your research. If you are not sure, read the appropriate sections before making your selection.

☒ Life sciences ☐ Behavioural & social sciences ☐ Ecological, evolutionary & environmental sciences

For a reference copy of the document with all sections, see nature.com/authors/policies/ReportingSummary-flat.pdf

Life sciences study design

All studies must disclose on these points even when the disclosure is negative.

Sample size	The sample size for the fMRI study was chosen based on a previous experiment (Benoit, Szpunar, & Schacter, PNAS, 2014) using a similar design. The sample size for the replication study was determined (and pre-registered) to provide 80% power to detect an effect size of 2/3 the original effect found in the fMRI study.
Data exclusions	Twelve participants of the fMRI study had to be excluded either because of falling asleep in the scanner (two) or excessive head movements (ten). Excessive head movement was defined as maximal absolute motion > 3 mm or more than 5 individual movements > 0.5 mm in any functional run.
Replication	We conducted a pre-registered replication study (https://aspredicted.org/9ti3h.pdf) that corroborated the reproducibility of the simulation-induced attitude change.
Randomization	Both studies used a within-participant manipulation, hence they did not require randomization into groups.
Blinding	Both studies used a within-participant manipulation, hence they did not require randomization into groups that investigators could have been blinded to. In addition, all tasks were administered electronically.

Reporting for specific materials, systems and methods

Materials & experimental systems

n/a	Involved in the study	
☒	☐	Unique biological materials
☒	☐	Antibodies
☒	☐	Eukaryotic cell lines
☒	☐	Palaeontology
☒	☐	Animals and other organisms
☐	☒	Human research participants

Methods

n/a	Involved in the study	
☒	☐	ChIP-seq
☒	☐	Flow cytometry
☐	☒	MRI-based neuroimaging

Human research participants

Policy information about studies involving human research participants

Population characteristics	All included participants reported no history of psychiatric or neurological disorders.<br>fMRI study: 18 participants (3 male), mean age: 21.33 y, range: 18 – 27 y, all right handed, native English speakers; Replication study: 30 participants (17 male), mean age: 23.97 y, range: 20 – 32 y, native German speakers
Recruitment	For the fMRI study, we recruited participants via advertisements in the communities of Harvard and Boston University. For the replication study, we recruited via the participant database of the Max Planck Institute for Human Cognitive and Brain Sciences.

Magnetic resonance imaging

Experimental design

Design type	Task fMRI with event-related design.
Design specifications	The reported MRI data were acquired in seven function runs, each of which lasted for 440s (~7.5 min). Each run was comprised of 28 trials that started with a 2 s fixation cross, continued with the respective critical task for 7.5 s and a subsequent rating for a max. duration of 3 s. The remainder of the maximal response time plus 3 s constituted the fixed ITI which was extended by a jittered period (0 to 8 s in 2 s intervals). Participants imagined individual people and places in runs 1, 2, 6, and 7 and interactions with people at specific places in runs 3 to 5.
Behavioral performance measures	After each individual trial, participants rated the vividness of the imagined episode on a five point scale.

Acquisition

Imaging type(s)	functional
Field strength	3 Tesla
Sequence & imaging parameters	Functional images were acquired with a T2*-weighted echo-planar imaging (EPI) pulse sequence that employed multiband RF pulses and Simultaneous Multi-Slice (SMS) acquisition with the following parameters: 69 interleaved axial-oblique slices (angled 17° towards coronal from AC-PC), TR=2000ms, TE=27ms, flip angle=80°, 2x2x2mm^2 voxels, 6/8 partial fourier, FOV=216mm, SMS=3.
Area of acquisition	whole brain

Diffusion MRI ☐ Used ☒ Not used

Preprocessing

Preprocessing software	The images were preprocessed using SPM12 in Matlab 2012a.
Normalization	Functional and structural images were normalized to MNI space using a 4th degree B-spline non-linear transformation.
Normalization template	ICBM space template - European brains
Noise and artifact removal	The realignment parameters were included in the first level GLMs.
Volume censoring	No censoring was performed.

Statistical modeling & inference

Model type and settings	For the parametric modulation analyses, we modeled all simulation periods with a single regressor and a boxcar function of 7.5 s duration plus additional regressors for the respective number of parametric modulators. For whole brain-analyses, individual contrast estimates were entered into a t-test at the second level. For RSA, we modeled each trial with a separate regressor.
Effect(s) tested	A first GLM estimated brain activation changes separately for each simulation during phases 1 and 3 (thus including 112 regressors, one for each of the two simulations of the 28 places and 28 people). The t-values of the ensuing parameter estimates entered the RSA.<br><br>A second GLM assessed brain activation changes associated with the affective value (i.e., liking) of the simulated people and places during phases 1 and 3. We therefore entered a regressor coding for the duration of all simulation trials plus an additional parametric regressor coding for the liking of the respective simulated item. Given that the integrative simulations during phase 2 changed attitudes, we used the pre-ratings for phase 1 and post-ratings for phase 3.<br><br>A third GLM assessed brain activation during phase 2. We entered (i) a regressor coding for all simulations, (ii) a first parametric modulator coding for the affective value of the UCS (i.e., liking of the person, averaged across pre- and post-ratings), and (iii) a second parametric modulator coding for the change in value of the CS (i.e., post- minus pre-rating liking of the place). Additional GLMs corroborated effects of affect-transfer, either without the first parametric modulator or with the first modulator controlling for the plausibility of the pairing. Two further GLMs controlled for the familiarity of the person, either by including it as a first modulator or by using residuals of the change scores after regressing out possible contributions of familiarity.

Specify type of analysis: ☐ Whole brain ☒ ROI-based ☐ Both

Anatomical location(s)	Following Liu, Grady, & Moscovitch (2017), an anatomical mask of our region-of-interest, the vmPFC, was created by merging the gyrus rectus and the medio-orbital section of the frontal gyrus of the Automatic Anatomical Labeling template using the WFU-Pickatlas toolbox.
Statistic type for inference (See Eklund et al. 2016)	For additional exploratory whole-brain analyses, results are reported at $p < 0.05$, FWE-cluster corrected with a cluster forming threshold of $p < 0.001$ and at least 15 contiguous voxels.

Correction

family-wise error correction

Models & analysis

n/a	Involved in the study
☒	☐ Functional and/or effective connectivity
☒	☐ Graph analysis
☒	☐ Multivariate modeling or predictive analysis
