# Supplementary material for: Forming attitudes via neural activity supporting affective episodic simulations
Source: Nat Commun. 2019 May 17;10:2215. doi: 10.1038/s41467-019-09961-w (PMC6525197; doi:10.1038/s41467-019-09961-w)
Supplement: Supplementary file 2 — Peer Review [file 41467_2019_9961_MOESM2_ESM.pdf]

Reviewers' comments:

Reviewer #1 (Remarks to the Author):

This well-written manuscript describes an elegant and creative study with results that will be interesting to a broad audience. It offers a compelling connection between the somewhat separate literatures focussed on the role of vmPFC in affect, value, and memory. The study was well designed, the analyses are appropriate and very clearly described, and the conclusions are well supported by the data.

I have only a few comments and questions to offer:

1. Do you find a parallel effect in the hippocampus? Given prior work suggesting a role for the hippocampus in transfer of value, I wonder whether you looked in a hippocampal ROI, and/or whether you find connectivity between the hippcocampus and vmPFC related to transfer of affective value.

2. Some of the figures have multiple components which are not always easy to map to the description in the caption. Maybe it would help to have more clearly labeled subcomponents. E.g. Figure 3 and Figure 4.

3. Figure 1, panel b: are these model predictions or raw data? If raw data, why no error bars? It would be useful to see the variability on the pre and post, and not just on their delta.

4. It would be helpful to state the final sample size for each study with the first description of the results.

5. For the RSA analyses, to what extent could the affective signal be biasing the between-within comparison? That is, it seems likely that affect varies more across items than within items, and it is also known that affect accounts for much of the variability in the vmPFC signal, so could this alone explain the between vs. within difference? It seemed that affect was not included the original RSA analysis, if I understood correctly.

6. Having to exclude 12 participants from a 30 participant sample is unusually high. Any thoughts on why this happened and whether it is something specific to this study for any reason? It may be good to also take this on in the discussion preemptively; in particular, to state that despite the low sample size you are left with, you have the power to explain the variability in behavior. This seems a strength of the paper that could be made more explicit.

Reviewer #2 (Remarks to the Author):

Key results: Benoit et al investigated whether affective simulation can alter value representations. To do so, the authors used a naturalistic place-person paradigm and behaviourally showed (+ replicated) that simulations involving liked (versus disliked) people in conjunction with a neutral location led to a subsequent increase in the like-ability of the location. Neurally, the author's found evidence that supports they hypothesis that such a transfer of value (ToV) effect is mediated by the vMPFC. This was a clear, interesting paper. To the best of my judgment, the behavioral and fMRI methods and results (including the RSA) appear valid and robust with appropriate control analyses in place. The methods and results are reported in sufficient detail. Below are specific comments for the authors that I hope will be considered by the authors.

1. The vMPFC and ToV

Can the author's align their work with other studies on transfer of value effects (+vmPFC) more broadly? For example, memory research involving laboratory items attributes ToV effects to the striatum + hippocampus in addition to vMPFC (i.e., studies from the Shohamy lab). Moreover, the reappraisal literature, in which participants are trained to generate positive frames for negative experiences, emphasize vmPFC -subcortical interactions in mediating such effects. vmPFC thus appears to be important for ToV within memory and imagination, along with other regions. Can the authors comment a bit more under what circumstances vmPFC may be more versus less relevant and how the vMPFC might be liaising with other regions to support ToV effects (and under what circumstances). I understand the authors are focused specifically on vMPFC in this paper but it would be helpful to place this work into a larger context. (Perhaps even referencing caudate effects reported in supplementary materials; Table S4. or caudate, and MTL associations with anticipated pleasantness in Benoit et al.) This could also tie into the authors' comment about the vMPFC computing a prediction error that subsequently gets integrated/updated—does this all happen in vMPFC? As a related point, I was intrigued that the parametric modulation effects in the present study appeared stronger for pre versus post (figure 3), although this is not verified statistically. I wondered if newly updated values require more cross regional input before the values get consolidated in the vmPFC (or the new vmPFC-based value representations may be more 'noisy'). I do not expect the authors to be so speculative but it would be useful to discuss more broadly how vMPFC might update value WRT other relevant circuitry.

2. A consideration of valence.

I paused when the authors stated: "Somewhat surprisingly, we observed this positive change in liking also for places imagined with disliked people" because the authors did not actually state their prediction for the disliked people (unless I missed this). It seems that the authors treat this as a baseline condition, but did the authors expect the scenes paired with disliked people to become more negative (putting mere exposure effects aside)? It would be helpful to be more clear. More broadly, I wondered whether the authors believe that the involvement of the vMPFC in 'emergent value' would be valence specific. Data from Roy et al. (who the authors cite) may suggest that these effects may be stronger for positive affect, although other literature may not support this idea. In any case, can the authors unpack their predictions a bit more, especially considering the authors refer to clinical populations (wherein both positive and negative valence alterations are relevant) in the discussion?

3. Processes involved in TOV

ToV effects are often thought to be implicit (e.g., Wimmer and Shohamy, 2012). In the present study, I was curious as to whether participants were aware of the change in their ratings and if so, were they aware that the changes were due to the pairing of the people and places? An explicit effect does not dampen my enthusiasm for the current findings but this information may be of interest to readers of this work if the authors have this information available. As an aside, I was also curious about whether the TOV effects could conceivably go in both directions (here the authors show that places become more + or – after exposure to liked versus disliked people but do those people become more neutral having been paired with neutral scenes?). The present data do not allow for a proper test of this idea but it would be an interesting effect, if observed.

4. The adaptive value of simulation

I thought that the author's discussion of the role of simulation in fostering more farsighted decisions was interesting (lines 304-309). I just have a few thoughts about one point: The authors suggest that exaggerated simulations can produce a distorted world model that can go awry, as in some clinical populations. However, it is also true that exaggerated simulations (for example overestimating the positive value of the future) can simply be adaptive, as it can help us counteract the temptation to

discount future rewards by making the future more affectively salient. Likewise, even overestimating negative future value can be useful to the extent that it leads to avoidance of possibly impending threats. Of course this can all go awry but exaggerated simulations can largely be adaptive and are ubiquitous phenomena in humans (as in impact and affective biases). Some of the ideas are discussed in a review by Bulley et al.

Minor

In the legend, please denote what the black dots refer to in Figure 2d (assumed to be outliers but helpful to state).

Repetition in phase 2 is noted but can the authors elaborate there about why Ss were asked to imagine the integrated experience 3x and how this was determined.

For clarity, When stating: "In the following, we first establish whether simulated episodes, similar to actual encounters, can shape real-life attitudes by reporting the results of an fMRI study and a preregistered replication," I would add "behavioral" ('the behavioral results of an fMRI study').

I have observed a bit of pushback in reporting sub-clusters when employing cluster correction methodology (although I too have reported sub-clusters).

E.g., Wan-Woo and collaborators state:
"Popular software packages such as SPM, FSL, and AFNI include algorithms for identifying multiple “peak” activations within large clusters and reporting a series of coordinates. However, these “peaks” cannot be used to infer that all of the “peaks” in the table are truly activated. In addition, it cannot be assumed that the coordinates listed in the table are good estimates of the true peak activation locations. Therefore, a single descriptor that covers all the suprathreshold voxels in the cluster should be used, as it more accurately reflects the spatial uncertainty inherent in the results."

Although the decision to report the single descriptor versus sub-clusters holds less relevance here since vMPFC comes out as the main peak, I wanted to make the authors aware of this (although I was confused by what “vmPFC; CN” means in table S4).

It would have been ideal if familiarity ratings were matched in the initial study but I am satisfied with how the reviewers dealt with this.

Since the authors treated the dislike condition as the baseline, it was less problematic that the initial ratings of the liked people were stronger in terms of valence from the midline than the disliked (e.g., 7.8 versus 3.2), although it would be more ideal to match these.

Reviewer #3 (Remarks to the Author):

In the manuscript entitled “Forming attitudes via neural activity supporting affective episodic simulations” the authors claim that imagination of persons in a spatial scene transfers value, as measured by likability ratings, from a person to a scene. They find the vmPFC to represent persons and scenes and observed value change related activity in the vmPFC. Overall, I think that this an interesting effect presented in a well-written manuscript.

However, I have a concern regarding the interpretation. It appears to me that the effect might be driven by familiarity. First of all, as liked people are more familiar than disliked people, it would be great to report the extent of the correlation between likability and familiarity. If the interpretation of

the findings is just about value, I'm concerned that the value transfer seemed to be only working for positive values. If the finding would be about value than I would expect a symmetric effect of opposite direction for disliked people, as value can be negative. In contrast, familiarity cannot be negative, so it seems to be the more simple explanation. If the authors want to stick to the value interpretation, I think familiarity should at least be included in the analysis. Familiarity could be regressed out of the behavioral effect and be included as a first parametric modulator in the fMRI GLM analysis. As this is crucial for the interpretation, I think it is important to rule out this alternative explanation.

Also, I'm wondering why the hippocampus is not part of the analysis, as previous studies like Wimmer et al. 2012 showed an involvement in value transfer. This should be discussed at minimum.

Last, I'm curious how the transfer might be happening. I was hoping for more RSA related analysis to describe the new link between persons and scenes. This might be worth testing and reporting (even for null findings)?

Minor points:
- Abstract line 14 is misleading as not all elements of the episodic simulation are manipulated. Episodic simulations do seem to only transfer from persons to scenes as the reverse effect is not observed or tested
- Figure 1: it would be great to list all ratings done at least in the caption
- Figure 3: as only neutral scenes are used this is, in fact, a correlation of person's likability but not scene's likability? Would be great to see this separately for both categories.

REPLIES TO REVIEWERS' SUGGESTIONS

Reviewer #1 *(Remarks to the Author):*

This well-written manuscript describes an elegant and creative study with results that will be interesting to a broad audience. It offers a compelling connection between the somewhat separate literatures focussed on the role of vmPFC in affect, value, and memory. The study was well designed, the analyses are appropriate and very clearly described, and the conclusions are well supported by the data.

I have only a few comments and questions to offer:

Reviewer's comment: *1. Do you find a parallel effect in the hippocampus? Given prior work suggesting a role for the hippocampus in transfer of value, I wonder whether you looked in a hippocampal ROI, and/or whether you find connectivity between the hippcocampus and vmPFC related to transfer of affective value.*

Our response: Indeed, the cited study by Wimmer & Shohamy (2012) has implicated the hippocampus in the transfer of value and thus may conceivably suggest a contribution of this region to the observed attitude change. However, we had more specifically hypothesized an involvement of the vmPFC in transferring value. This hypothesis was based on the idea that this region is particularly involved in coding for schematic representations of familiar elements from our environment. By contrast, we had reasoned that the hippocampus may be more important if value is transferred between associations of completely novel items (as in Wimmer & Shohamy, 2012). Consistent with this expectation, our exploratory whole-brain analysis had not provided any evidence for a hippocampal contribution to the transfer of value. We have now supplemented our result section with the additional region-of-interest analysis of the hippocampus, which also yielded a null effect (t_{17} = 0.04, p = 0.97) (from p. 12, 2nd paragraph; p. 21, 4th paragraph). We also take this comment as an opportunity to explicitly discuss possible dissociable roles for the vmPFC versus the hippocampus in the context of transferring value and inducing attitude change (from p. 15, 3rd paragraph).

Finally, inspired by the reviewer's suggestion, we conducted an exploratory psychophysiological interaction analysis seeded in the vmPFC. However, this analysis did not provide any clear-cut evidence (i.e., essentially null effects). Due to the explorative nature of this analysis and their inconclusive results, we feel that it would not add much to the manuscript.

Reviewer's comment: *2. Some of the figures have multiple components which are not always easy to map to the description in the caption. Maybe it would help to have more clearly labeled subcomponents. E.g. Figure 3 and Figure 4.*

Our response: Based on this suggestion, we have changed the layout of the figures to more clearly demarcate the individual panels. The revised figures 3 and 4 now also include clear labels for the region-of-interest versus whole-brain analyses.

On a related matter, we also realized that we had swapped the labels for "disliked" and "liked" in the bottom panel of Fig. 2, which we have now corrected (with no consequence for the interpretation). We sincerely apologize for this mistake.

Reviewer's comment: *3. Figure 1, panel b: are these model predictions or raw data? If raw data, why no error bars? It would be useful to see the variability on the pre and post, and not just on their delta.*

Our response: These are indeed data from the experiments. Given that the inferential statistics were based on the difference scores, we had chosen to display error bars only for these values. However, we agree with the reviewer that it is always best to visualize the data as comprehensively as possible, and we were therefore happy to amend the figure accordingly.

Reviewer's comment: *4. It would be helpful to state the final sample size for each study with the first description of the results.*

Our response: We think this is good advice, particularly given that the methods are only presented at the end of the manuscript. In the revised version, we thus state the sample sizes of the two studies in the very first paragraph of the result section (p. 5, 2nd paragraph).

Reviewer's comment: *5. For the RSA analyses, to what extent could the affective signal be biasing the between-within comparison? That is, it seems likely that affect varies more across items than within items, and it is also known that affect accounts for much of the variability in the vmPFC signal, so could this alone explain the between vs. within difference? It seemed that affect was not included the original RSA analysis, if I understood correctly.*

Our response: Indeed, we report that affective value accounts for variability in the univariate vmPFC BOLD signal and agree that it's important to thoroughly examine possible consequences for the interpretation of the RSA analysis. Therefore, the revised manuscript explicitly discusses this potential caveat, explains how our original analyses addressed this issue, and includes an additional control analysis (as summarized below).

First, we would suggest that our original analysis had already accounted for most of the variance due to value. That is, the *between-item similarity* measures were based on items of only the same material (people or places) and, critically, of only the same valence (liked or disliked) as the respective *within-item*

measures. This approach ensures that a smaller *between-* than *within-item similarity* cannot merely reflect greater variability due to categorical differences in affect (as may have been the case if the *between-item similarity* had included items of the opposite valence). Our analysis is thus not susceptible to this putative source of variance in vmPFC pattern information (see discussion on p. 8, 1st paragraph).

In addition, we have now performed a complementary analysis of the pattern information, which we refer to in the revised Results (p. 9, 1st paragraph) and describe in detail in the Supplement (Supplemental Fig. 2). Specifically, this analysis does not broadly examine *between-item similarity* by comparing the activity pattern for a given item with the activity patterns of all other items of the same category (e.g., liked people). Instead, for each item, we identified one item (of the same category and from the same functional run) that, importantly, had received the identical liking rating in the post test. We thus ensure that the comparison of the item with itself (*within-item similarity*) and with its paired item (*matched-liking similarity*) is exactly matched in terms of value. The analysis is based on the 78.08 % of items for which it was possible to assign such a match.

However, for many items, there was more than one possible match. Therefore, on each of 1000 iterations, we randomly drew one of the possible matches as a partner before then computing the similarity between the items and their respective partners. We finally averaged these similarity scores across all iterations, which we took as an estimate of the *matched-liking similarity*. Critically, this approach yielded the predicted larger *within-item* than *matched-liking similarity* ($F_{1,17} = 14.47$, $p = 0.001$, $\eta^2 = 0.46$), i.e., a greater *within-item* similarity even when we directly match the similarity measures on variability in value. The control analysis thus further demonstrates that the vmPFC codes for individual elements from our environment.

Reviewer's comment: *6. Having to exclude 12 participants from a 30 participant sample is unusually high. Any thoughts on why this happened and whether it is something specific to this study for any reason? It may be good to also take this on in the discussion preemptively; in particular, to state that despite the low sample size you are left with, you have the power to explain the variability in behavior. This seems a strength of the paper that could be made more explicit.*

Our response: Indeed, we had to exclude more participants than usual due to excessive motion. We don't have a definitive answer as to its cause. However, we scanned this project shortly before the MRI scanner was updated from a *TimTrio* to a *Prisma*. This made it a busy time at the *Center for Brain Science* with lots of projects being wrapped up at the same time. We thus had to schedule participants also outside of the regular hours (i.e., early mornings and late evenings). Maybe our participants found it particularly difficult to remain still during those times. However, as suggested, the revised manuscript now explicitly emphasizes that we had obtained a behavioral effect that we have clearly replicated in a larger sample with a similar effect size in the pre-registered study (p. 16, 2nd paragraph). (We also would like to mention that, in the meantime, we have successfully replicated key fMRI results in a follow-up project.)

***Reviewer #2** (Remarks to the Author):*

Key results: Benoit et al investigated whether affective simulation can alter value representations. To do so, the authors used a naturalistic place-person paradigm and behaviourally showed (+ replicated) that simulations involving liked (versus disliked) people in conjunction with a neutral location led to a subsequent increase in the like-ability of the location. Neurally, the author's found evidence that supports they hypothesis that such a transfer of value (ToV) effect is mediated by the vMPFC. This was a clear, interesting paper. To the best of my judgment, the behavioral and fMRI methods and results (including the RSA) appear valid and robust with appropriate control analyses in place. The methods and results are reported in sufficient detail. Below are specific comments for the authors that I hope will be considered by the authors.

Reviewer's comment: *1. The vMPFC and ToV*

Can the author's align their work with other studies on transfer of value effects (+vmPFC) more broadly? For example, memory research involving laboratory items attributes ToV effects to the striatum + hippocampus in addition to vMPFC (i.e., studies from the Shohamy lab). Moreover, the reappraisal literature, in which participants are trained to generate positive frames for negative experiences, emphasize vmPFC -subcortical interactions in mediating such effects. vmPFC thus appears to be important for ToV within memory and imagination, along with other regions. Can the authors comment a bit more under what circumstances vmPFC may be more versus less relevant and how the vMPFC might be liaising with other regions to support ToV effects (and under what circumstances). I understand the authors are focused specifically on vMPFC in this paper but it would be helpful to place this work into a larger context. (Perhaps even referencing caudate effects reported in supplementary materials; Table S4. or caudate, and MTL associations with anticipated pleasantness in Benoit et al.) This could also tie into the authors' comment about the vMPFC computing a prediction error that subsequently gets integrated/updated—does this all happen in vMPFC? […] *I do not expect the authors to be so speculative but it would be useful to discuss more broadly how vMPFC might update value WRT other relevant circuitry.*

Our response: Though our study focuses on the contribution of the vmPFC, we did not mean to diminish the possible roles of the striatum and hippocampus in transferring value. We have thus taken this comment as an opportunity to considerably extend our discussion on this topic (from p. 15, 2nd paragraph). In the revised manuscript, we refer to the extant literature that has associated the striatum with the transfer of value form a UCS to a CS (see Shohamy, 2011). Together with our finding that striatal activity tracks the value of the imagined UCS, we take this to suggest that the striatal value signal may be conveyed to the vmPFC. In this region, it could then interact with the existing schematic representations of the CS to process an updated value estimate.

With respect to the hippocampus, we argue that previous studies have focused on the transfer of value between arbitrary combinations of novel stimuli (Wimmer & Shohamy, 2012; Gilboa et al., 2014). We suggest that such situations require the kind of rapid encoding that is afforded by this region (Eichenbaum & Cohen, 2014; Kumaran, Hassabis, & McClelland, 2016; see also Wimmer, Daw, & Shohamy, 2012). By contrast, the current study examined changes in attitude towards well-established elements from participants' real life environment. As such, the integrative simulations could be supported by the co-activation of established knowledge structures that are already represented in the vmPFC (see Benoit et al., 2014). Therefore, these simulation-induced attitude changes may be less reliant on hippocampal processes than more episodic-like forms of value transfer (as in Wimmer & Shohamy, 2012).

We conclude by arguing that it will be an important avenue for future studies to systematically delineate the individual contributions of the striatum, hippocampus, and vmPFC as well as their interactions (Shohamy, 2015; Gerraty et al., 2014).

Please note that we do not make references to the reappraisal literature in this context, because we understand that the suggested role of the vmPFC did not survive meta-analytical scrutiny (Buhle et al., *Neuroimage*, 2014). However, if we have overlooked pertinent evidence, we'd be happy to learn about the respective articles.

Reviewer's comment: *As a related point, I was intrigued that the parametric modulation effects in the present study appeared stronger for pre versus post (figure 3), although this is not verified statistically. I wondered if newly updated values require more cross regional input before the values get consolidated in the vmPFC (or the new vmPFC-based value representations may be more 'noisy').*

Our response: We think that these are intriguing ideas. However, we are reluctant to make much of the observation that the effect sizes of the parameter estimates are numerically lower in *phase III* than *phase I*. Previous work has shown that repeated simulations of the same episode can lead to reduced activity in critical core-network regions including the vmPFC (Szpunar et al., *Soc. Cogn. Affect. Neurosci*, 2014). A possible reduction of the parametric effect may therefore simply reflect a similar mechanism of repetition suppression.

Reviewer's comment: *2. A consideration of valence.*

I paused when the authors stated: "Somewhat surprisingly, we observed this positive change in liking also for places imagined with disliked people" because the authors did not actually state their prediction for the disliked people (unless I missed this). It seems that the authors treat this as a baseline condition, but did the authors expect the scenes paired with disliked people to become more negative (putting mere exposure effects aside)? It would be helpful to be more clear.

Our response: The reviewer is correct that we had primarily expected a more positive change in attitudes for places imagined with liked people. As such, the places imagined with disliked people served as a baseline to establish this effect. However, our hypothesis is also consistent with a negative change in liking following the simulation of negative experiences (please see also reply to the following comment). As discussed, in the current study, such a negative change may have been masked by simple effects such as mere exposure that would nudge attitudes in the opposite, positive direction. We have clarified this point in the revised discussion (p. 16, 3rd paragraph). Indeed, in future work, we intend to follow-up on this observation by including a neutral condition (e.g., imaginings with neutral people) that will allow us to further characterize the relative change in attitude.

Reviewer's comment: *More broadly, I wondered whether the authors believe that the involvement of the vMPFC in 'emergent value' would be valence specific. Data from Roy et al. (who the authors cite) may suggest that these effects may be stronger for positive affect, although other literature may not support this idea. In any case, can the authors unpack their predictions a bit more, especially considering the authors refer to clinical populations (wherein both positive and negative valence alterations are relevant) in the discussion?*

Our response: Good point. The vmPFC would arguably also have to code for different degrees of "negative value" for this mechanism to also induce negative shifts in clinical populations. Indeed, this is consistent with our understanding of the extant literature. The value signal in the vmPFC seems to be best understood as coding for a single dimension that monotonically increases from negative over neutral to positive (rather than as coding for salience or arousal) (Barta et al., 2013; Litt et al., 2011). Accordingly, we suggest that the contribution of the vmPFC to processing value of simulated events is not valence specific. Rather it may more generally signal different degrees of the presence (or absence) of value. Therefore, the same mechanism may mediate not just positive but also negative shifts in attitude. We now explicitly discuss this argument in the revised manuscript (p. 14, 3rd paragraph; p. 15, 1st paragraph).

Reviewer's comment: *3. Processes involved in TOV*

ToV effects are often thought to be implicit (e.g., Wimmer and Shohamy, 2012). In the present study, I was curious as to whether participants were aware of the change in their ratings and if so, were they aware that the changes were due to the pairing of the people and places? An explicit effect does not dampen my enthusiasm for the current findings but this information may be of interest to readers of this work if the authors have this information available.

Our response: We haven't collected any data that speak directly to this question. However, the observed link between the magnitude of attitude change and vmPFC signal indicates that the change was likely induced during the simulations rather than during the final test. Given that participants were not aware of

a final rating, we think that they were unlikely to engage in deliberate revaluation at that stage. We have added this reasoning to the discussion (p. 16, 2nd paragraph).

Reviewer's comment: *As an aside, I was also curious about whether the TOV effects could conceivably go in both directions (here the authors show that places become more + or – after exposure to liked versus disliked people but do those people become more neutral having been paired with neutral scenes?). The present data do not allow for a proper test of this idea but it would be an interesting effect, if observed.*

Our response: We had considered that such an outcome would be consistent with our hypothesis and indeed we have observed this pattern (i.e., liked and disliked people becoming more neutral) in both studies. However, we are hesitant to take this as additional support for our hypothesis, because the studies were not designed to include a proper baseline to evaluate these concordant changes in liking of the people. Therefore, we can't rule out simpler explanations for this effect such as regression to the mean (i.e., from either very positive or very negative to the neutral "mean"). However, we are of course happy to include these additional results in the revised Supplement (p. 6, 3rd paragraph; Supplemental Fig. 1).

Reviewer's comment: *4. The adaptive value of simulation*

I thought that the author's discussion of the role of simulation in fostering more farsighted decisions was interesting (lines 304-309). I just have a few thoughts about one point: The authors suggest that exaggerated simulations can produce a distorted world model that can go awry, as in some clinical populations.

However, it is also true that exaggerated simulations (for example overestimating the positive value of the future) can simply be adaptive, as it can help us counteract the temptation to discount future rewards by making the future more affectively salient. Likewise, even overestimating negative future value can be useful to the extent that it leads to avoidance of possibly impending threats. Of course this can all go awry but exaggerated simulations can largely be adaptive and are ubiquitous phenomena in humans (as in impact and affective biases). Some of the ideas are discussed in a review by Bulley et al.

Our response: We agree that episodic simulations can convey the adaptive benefit of making decisions more farsighted, and that this may even be the case for exaggerated simulations. We already had alluded to this point in the discussion with reference to our earlier work on simulation and discounting (Benoit et al., 2011). In the revised manuscript, we have extended this section (p. 16, 3rd paragraph) and amended our final paragraph (p. 17, 1st paragraph) by also referring to some of the interesting thoughts provided by Bulley et al. (2017) on the benefits of simulating unlikely yet costly threat events. We also include a reference to Miloyan and Suddendorf (2015), who more generally argue for the adaptive benefits of

exaggerated simulations and ensuing biases. We think that these edits have helped to balance the discussion of positive versus negative consequences of exaggerated simulations.

Minor

Reviewer's comment: *In the legend, please denote what the black dots refer to in Figure 2d (assumed to be outliers but helpful to state).*

Our response: Yes, these are indeed outliers. We have clarified this in the revised captions.

Reviewer's comment: *Repetition in phase 2 is noted but can the authors elaborate there about why Ss were asked to imagine the integrated experience 3x and how this was determined.*

Our response: In designing the procedure, we aimed to include multiple repetitions to foster the impact that simulations can exert on people's attitudes. However, at the same time, we also had to trade this off with the number of episodes that participants are able to simulate in a given session without getting overly fatigued. Piloting indicated that three repetitions were thus adequate in the sense (i) that participants could perform the task and (ii) that it was sufficient to yield the behavioral effect. We have described this in the revised methods (p. 19, 1st paragraph). However, for future studies, it will be interesting to manipulate the number of repetitions to examine possible "dosage" effects.

Reviewer's comment: *For clarity, When stating: "In the following, we first establish whether simulated episodes, similar to actual encounters, can shape real-life attitudes by reporting the results of an fMRI study and a preregistered replication," I would add "behavioral" ('the behavioral results of an fMRI study').*

Our response: We have implement this clarifying suggestion (p. 5, 2nd paragraph).

Reviewer's comment: *I have observed a bit of pushback in reporting sub-clusters when employing cluster correction methodology (although I too have reported sub-clusters).*

E.g., Wan-Woo and collaborators state:

"Popular software packages such as SPM, FSL, and AFNI include algorithms for identifying multiple "peak" activations within large clusters and reporting a series of coordinates. However, these "peaks" cannot be used to infer that all of the "peaks" in the table are truly activated. In addition, it cannot be assumed that the coordinates listed in the table are good estimates of the true peak activation locations. Therefore, a single descriptor that covers all the suprathreshold voxels in the cluster should be used, as it more accurately reflects the spatial uncertainty inherent in the results."

Although the decision to report the single descriptor versus sub-clusters holds less relevance here since vMPFC comes out as the main peak, I wanted to make the authors aware of this (although I was confused by what "vmPFC; CN" means in table S4).

Our response: We think it is a good idea to be explicit about this caveat in interpreting individual "peaks" in the tables, particularly following cluster correction. The revised captions thus include an appropriate cautionary note with reference to Woo et al. (2014).

Reviewer's comment: *It would have been ideal if familiarity ratings were matched in the initial study but I am satisfied with how the reviewers dealt with this.*

Since the authors treated the dislike condition as the baseline, it was less problematic that the initial ratings of the liked people were stronger in terms of valence from the midline than the disliked (e.g., 7.8 versus 3.2), although it would be more ideal to match these.

Our response: We agree.

Reviewer #3 *(Remarks to the Author):*

In the manuscript entitled "Forming attitudes via neural activity supporting affective episodic simulations" the authors claim that imagination of persons in a spatial scene transfers value, as measured by likability ratings, from a person to a scene. They find the vmPFC to represent persons and scenes and observed value change related activity in the vmPFC. Overall, I think that this an interesting effect presented in a well-written manuscript. However, I have a concern regarding the interpretation. It appears to me that the effect might be driven by familiarity.

Reviewer's comment: *First of all, as liked people are more familiar than disliked people, it would be great to report the extent of the correlation between likability and familiarity.*

Our response: We think it is a good idea to further describe the relationship between liking and familiarity in the fMRI study. We thus report the *Pearson* correlation between those values across liked and disliked people (mean $r = 0.58$; $SD = 0.31$) (p. 18, 2nd paragraph). To further characterize the association between familiarity and liking, we also complement the inferential statistics by reporting the numerical difference in familiarity ratings for the two conditions (from p. 5, 3rd paragraph).

Reviewer's comment: *If the interpretation of the findings is just about value, I'm concerned that the value transfer seemed to be only working for positive values. If the finding would be about value than I would expect a symmetric effect of opposite direction for disliked people, as value can be negative. In contrast, familiarity cannot be negative, so it seems to be the more simple explanation. If the authors want to stick to the value interpretation, I think familiarity should at least be included in the analysis. Familiarity could be regressed out of the behavioral effect and be included as a first parametric modulator in the fMRI GLM analysis. As this is crucial for the interpretation, I think it is important to rule out this alternative explanation.*

Our response: We thank the reviewer for this comment and the suggestions, which we have taken as an opportunity to further strengthen our interpretation. We completely agree that it is important to rule out the alternative explanation that the observed behavioral and fMRI effects are based on differences in familiarity rather than liking. In the revised manuscript, we therefore provide additional discussion – also of our previous analyses – and report the suggested complementary control analyses as detailed below.

Before describing the additional analyses, however, we first would like to mention that we had already provided evidence that renders the alternative *familiarity-account* unlikely. Regarding the behavioral effect, we had successfully replicated the change in attitude in the pre-registered replication study in which we had matched the disliked and liked people on familiarity. At least in that study, the effect can thus not have been caused by differences in familiarity. Regarding the fMRI effect, we already had included the suggested control analysis that included familiarity as the first parametric regressor. (However, we appreciate that this may have been somewhat hidden in the supplement.) We now more explicitly motivate and highlight this analysis in the result section (from p. 12, 1st paragraph).

Based on the reviewer's suggestions, we have also performed additional control analyses. We can confirm that these further corroborated our interpretation. In particular, as suggested, we determined whether the behavioral effect in the fMRI study remains significant when regressing out the effect of familiarity. For each participant, we therefore computed the residuals of the change in liking after regressing out the contribution of person familiarity. We then compared these residual change scores across conditions and indeed observed that there was still a stronger increase in liking for places that had been paired with liked than with disliked people (using a Wilcoxon test: $W_{17} = 143$, $p = 0.005$, matched rank biserial correlation $r = 0.67$, because of deviation from normality as indicated by Shapiro-Wilk: $W = 0.831$, $p = 0.004$) (p. 6, 4th paragraph). Consistent with the replication study, the behavioral effect could thus not be accounted for by differences in familiarity.

Moreover, we also used the residual scores to conduct an additional parametric modulation analysis of the fMRI data. This analysis identifies regions where the activation was modulated by the change in liking even after taking out the putative contribution of person familiarity. Consistent with our previous results, we obtained a significant effect in the vmPFC in this complementary control analysis (using a Wilcoxon test: $W_{17} = 136$, $p = 0.027$, matched rank biserial correlation $r = 0.59$, because of deviation from normality as indicated by Shapiro-Wilk: $W = 0.88$, $p = 0.029$) (p. 12, 1st paragraph; Supplemental Fig. 3).

Reviewer's comment: *Also, I'm wondering why the hippocampus is not part of the analysis, as previous studies like Wimmer et al. 2012 showed an involvement in value transfer. This should be discussed at minimum.*

Our response: We agree that this issue merits further clarification. We specifically had hypothesized an involvement of the vmPFC, based on this region's dual contribution to the representation of schemas (Milivojevic et al., 2015; Richter et al., 2016; Schlichting et al., 2015; Gilboa, & Marlatte, 2017) and the processing of value (Barta et al., 2013). That is, in our study, we examine changes in attitude towards places that participants were familiar with from their everyday environment. As shown in our previous work (Benoit et al., 2014), simulations including such familiar places are supported by the vmPFC, consistent with this region's involvement in representing schemas.

This is a critical difference to the studies that have implicated the hippocampus in value transfer (Wimmer & Shohamy, 2012; Gilboa et al., 2014). These studies have examined the transfer of value between novel abstract figures and their arbitrary associations. These situations thus likely required rapid encoding and binding processes that are supported by the hippocampus (Eichenbaum & Cohen, 2014; Kumaran, Hassabis, & McClelland, 2016; see also Wimmer, Daw, & Shohamy, 2012). We thus would argue that the transfer-of-value in our procedure more strongly engages schematic memory processes that are mediated by the vmPFC while the transfer in Wimmer and Shohamy (2012) more strongly relies on episodic memory processes that are mediated by the hippocampus. Though of course we believe that the hippocampus is generally important for simulations *per se*.

The revised manuscript includes an explicit discussion of this potential dissociation (from p. 15, 3rd paragraph). However, we agree that the extant literature warrants a closer look at the hippocampus and have thus included an additional region-of-interest analysis in the Results (from p. 12, 2nd paragraph; p. 21, 4th paragraph). Consistent with the whole-brain analysis, it yielded a clear null effect (t_{17} = 0.04, p = 0.97).

Reviewer's comment: *Last, I'm curious how the transfer might be happening. I was hoping for more RSA related analysis to describe the new link between persons and scenes. This might be worth testing and reporting (even for null findings)?*

Our response: The current study was designed to assess stability of representations within vmPFC (a finding that we are pleased to just have replicated in a follow-up project). We agree that it would be fascinating to also look at more subtle effects of changes in those representations (as in the elegant work by, e.g., Schlichting et al., *Nat Commun*, 2015, and Milivojevic et al., *Curr Biol*, 2015). However, to detect those smaller effects, we think that we would have had to further boost the reliability of the pattern estimate in the pre- and post-phase (for example, by including many more repetitions of each person and place).

Due to the already lengthy experiment, this was not feasible for the current project. As such, we think that the results (particularly null findings) would be inconclusive.

Minor points:

Reviewer's comment: *- Abstract line 14 is misleading as not all elements of the episodic simulation are manipulated. Episodic simulations do seem to only transfer from persons to scenes as the reverse effect is not observe*

Our response: In the revised manuscript, we also report the concordant changes in liking of the people (i.e., liked and disliked people becoming more neutral) (p. 6, 3rd paragraph; Supplemental Fig. 1). However, because the studies were not designed to examine this effect, they did not include a proper baseline that would have allowed us to rule out trivial explanations, such as simple regression to the mean (i.e., from either very positive or very negative to the neutral "mean"). We thus agree with the reviewer that we should precisely specify that episodic simulations had shaped attitudes towards the places. We have accordingly changed the final sentence of the abstract to limit our conclusions to those exactly justified by our results.

Reviewer's comment: *- Figure 1: it would be great to list all ratings done at least in the caption*

Our response: We completely agree and have amended the caption accordingly (Fig. 1).

Reviewer's comment: *- Figure 3: as only neutral scenes are used this is, in fact, a correlation of person's likability but not scene's likability? Would be great to see this separately for both categories.*

Our response: We agree with the reviewer that most variance in liking is due to the people, given that they entailed both liked and disliked exemplars. However, we would like to note that the parametric modulator does not just predict that liked and disliked people should show a strong difference in brain activation. Instead, it also predicts that activation for the neutral places should fall somewhere in the middle. We therefore think that the regressor picks up variance associated with a continuum of liking that spans both the people and the places. To enhance the clarity of the manuscript, we have amended the results (from p. 9, 3rd paragraph) and the figure caption (Fig. 3) to more carefully describe the assumptions of this analysis (including the possibly stronger contribution by the people).

Reviewers' comments:

Reviewer #1 (Remarks to the Author):

The authors were very responsive to the reviews, strengthening a paper that was strong to begin with. I recommend publication.

Reviewer #2 (Remarks to the Author):

The authors have done a very nice job with these revisions and I am very happy with the current version. This is an interesting, creative, and well written paper.

Two *very* minor comments:

1. p. 12: The discussion of the hippocampus in the paper feels abrupt: "However, previous studies have associated..."

I would consider a softer transition: e.g., "Beyond the vmPFC, previous studies..."

2. Figure S1 change "can’t" to "cannot"

Reviewer #3 (Remarks to the Author):

I would like to thank the authors for their answers and additional analysis. I’m happy that effects are still present after familiarity has been regressed out. Also, testing the involvement of the hippocampus is strengthening the manuscript in my opinion.

However, my concerns regarding the valence of value transfer are still persisting. All scenes are more liked after imagination. In my opinion, the interpretation of a value transfer is too far-fetched, as only positive values, but not negative ones are transferred. It is clearly working only for like persons and not for disliked persons (even the opposite is observed, as disliked persons induce more positive ratings in previously neutral places). Positive valence is only boosting the positive effect that is present for all stimuli.

If it would be a real value transfer, disliked persons should induce negative ratings for scenes (which is not the case as we see in Figure 1). Also, the fMRI analysis is just reflecting this increase in value and does not provide a deeper explanation. The important test there would be a separate analysis for like and disliked people, showing a change in vmPFC activity in opposite directions for liked and disliked people. Right now, I would suspect that the (directed) transfer is only present for liked people. Given the missing support of the behavioral data and unclear findings from the fMRI analysis, I find the claim of the paper too far-fetched. Often, “affective value” is used in the manuscript, which includes also negative values (for example line 20, 349, but also in many other sections as well). I would like to ask the authors to stick with an interpretation close to the results.

Please rephrase that this effect is only present in a positive direction.

REPLIES TO REVIEWERS' SUGGESTIONS

Reviewer #1 *(Remarks to the Author):*

The authors were very responsive to the reviews, strengthening a paper that was strong to begin with. I recommend publication.

Our response: We thank the reviewer for the positive assessment of the revisions and the manuscript.

Reviewer #2 *(Remarks to the Author):*

The authors have done a very nice job with these revisions and I am very happy with the current version. This is an interesting, creative, and well written paper.

*Two *very* minor comments:*
1. p. 12: The discussion of the hippocampus in the paper feels abrupt: "However, previous studies have associated..."
I would consider a softer transition: e.g., "Beyond the vmPFC, previous studies..."
2. Figure S1 change "can't" to "cannot"

Our response: We thank the reviewer for their positive evaluation of the revisions and resulting manuscript. We appreciate the stylistic suggestions, which we were happy to adopt in the latest version (p. 13, 1st paragraph; caption to Figure S1).

Reviewer #3 *(Remarks to the Author):*

I would like to thank the authors for their answers and additional analysis. I'm happy that effects are still present after familiarity has been regressed out. Also, testing the involvement of the hippocampus is strengthening the manuscript in my opinion.

Reviewer's comment: *However, my concerns regarding the valence of value transfer are still persisting. All scenes are more liked after imagination. In my opinion, the interpretation of a value transfer is too far-fetched, as only positive values, but not negative ones are transferred. It is clearly working only for like persons and not for disliked persons (even the opposite is observed, as disliked persons induce more positive ratings in previously neutral places). Positive valence is only boosting the positive effect that is present for all stimuli.*

If it would be a real value transfer, disliked persons should induce negative ratings for scenes (which is not the case as we see in Figure 1). Also, the fMRI analysis is just reflecting this increase in value and does not provide a deeper explanation. The important test there would be a separate analysis for like and disliked people, showing a change in vmPFC activity in opposite directions for liked and disliked people. Right now, I would suspect that the (directed) transfer is only present for liked people. Given the missing

support of the behavioral data and unclear findings from the fMRI analysis, I find the claim of the paper too far-fetched. Often, "affective value" is used in the manuscript, which includes also negative values (for example line 20, 349, but also in many other sections as well). I would like to ask the authors to stick with an interpretation close to the results.

Please rephrase that this effect is only present in a positive direction.

Our response: We agree that it is prudent to discuss the transfer of value more cautiously, and have accordingly amended the manuscript as recommended (please see below). In the previous version, we had already discussed the observation that simulations featuring disliked people also lead to a positive shift in attitude. We had suggested that this may have been caused by simple effects, such as mere exposure, that would boost the value of any imagined place and thus mask a possible downward shift (from p. 16, 4th paragraph). (Of course, the important finding is the *greater* positive change in liking for places paired with liked than with disliked people.)

However, we agree that a more cautious description and interpretation of this effect is warranted. We thus have carefully edited the manuscript in line with the reviewer's suggestion. Throughout the revised manuscript, we make explicit that the effect was always in the positive direction and that we obtained evidence for a transfer of positive value only (in the Abstract; Introduction: p. 5, 1st paragraph; Results: p. 13, 2nd paragraph; caption to Fig. 4; Discussion: p. 15, 2nd paragraph). Moreover, in the discussion, we reiterate this point to then conclude that it "remains to be determined whether this mechanism can also lead to a downward shift via a transfer of *negative* affective value" (p. 15, 2nd paragraph).
